# Tumor Microenvironment Responsive CD8^+^ T Cells and Myeloid‐Derived Suppressor Cells to Trigger CD73 Inhibitor AB680‐Based Synergistic Therapy for Pancreatic Cancer

**DOI:** 10.1002/advs.202302498

**Published:** 2023-10-22

**Authors:** Qiangda Chen, Hanlin Yin, Junyi He, Yuqi Xie, Wenquan Wang, Huaxiang Xu, Lei Zhang, Chenye Shi, Jun Yu, Wenchuan Wu, Liang Liu, Ning Pu, Wenhui Lou

**Affiliations:** ^1^ Department of Pancreatic Surgery Cancer Center Department of General Surgery Zhongshan Hospital Fudan University Shanghai 200032 China; ^2^ Departments of Medicine and Oncology Johns Hopkins University School of Medicine Baltimore MD 21287 USA

**Keywords:** CD73, AB680, combination therapy, pancreatic cancer

## Abstract

CD73 plays a critical role in the pathogenesis and immune escape in pancreatic ductal adenocarcinoma (PDAC). AB680, an exceptionally potent and selective inhibitor of CD73, is administered in an early clinical trial, in conjunction with gemcitabine and anti‐PD‐1 therapy, for the treatment of PDAC. Nevertheless, the specific therapeutic efficacy and immunoregulation within the microenvironment of AB680 monotherapy in PDAC have yet to be fully elucidated. In this study, AB680 exhibits a significant effect in augmenting the infiltration of responsive CD8^+^ T cells and prolongs the survival in both subcutaneous and orthotopic murine PDAC models. In parallel, it also facilitates chemotaxis of myeloid‐derived suppressor cells (MDSCs) by tumor‐derived CXCL5 in an AMP‐dependent manner, which may potentially contribute to enhanced immunosuppression. The concurrent administration of AB680 and PD‐1 blockade, rather than gemcitabine, synergistically restrain tumor growth. Notably, gemcitabine weakened the efficacy of AB680, which is dependent on CD8^+^ T cells. Finally, the supplementation of a CXCR2 inhibitor is validated to further enhance the therapeutic efficacy when combined with AB680 plus PD‐1 inhibitor. These findings systematically demonstrate the efficacy and immunoregulatory mechanism of AB680, providing a novel, efficient, and promising immunotherapeutic combination strategy for PDAC.

## Graphical abstract

The selective inhibitor of CD73, AB680, increases the infiltration of responsive CD8^+^ T cells by decreasing immunosuppressive adenosine level. AB680 also promotes the myeloid‐derived suppressor cell (MDSC) chemotaxis via tumor‐derived CXCL5 in an AMP‐dependent manner. Targeting the CXCL5/CXCR2 pathway with the CXCR2 inhibitor SB‐225002 disrupts the “side effect” of AB680 and potentiates the efficacy of AB680 plus anti‐PD‐1 therapy.

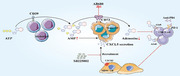

## 1 Introduction

Pancreatic ductal adenocarcinoma (PDAC), the predominant form of pancreatic cancer, represents a devastating malignancy characterized by a 5‐year survival rate of ≈12% and is predicted to be the second leading cause of cancer‐related mortality in the United States by the year 2030.^[^ ^1^ ^]^ Merely 20% of individuals diagnosed with PDAC are eligible for potentially curative surgical resection as most patients have progressed to advanced stages. Additional drug‐based therapy remains a crucial measure to prolong the lives of both inoperable patients with locally advanced or metastatic diseases, and those seeking to prevent recurrence following surgery.^[^ ^2^ ^]^ Over the past few years, a multitude of clinical trials have been initiated to screen for optimal regimens in an evidence‐based manner. Nevertheless, the peculiar tumor characteristics of PDAC present formidable obstacles in effective management, such as insensitivity or resistance to gemcitabine, which is the first‐line agent for PDAC chemotherapy.^[^ ^3^ ^]^ Furthermore, the absence of immune cell infiltration contributes to the failure of immunotherapy in PDAC as well, despite its glory in many other tumor entities.^[^ ^4^ ^]^ Thus, a more profound understanding of the intricate interactions in the PDAC microenvironment is imperative to explore novel potential therapeutic strategies to mitigate intra‐tumor adverse effects due to its complexity and heterogeneity.

Hypoxia, a prevalent characteristic observed in various solid cancers, has been acknowledged as an initiating factor for triggering metabolic reprogramming and immunological changes.^[^ ^5^ ^]^ The presence of numerous stromal cells and extracellular matrix, coupled with inadequate vascularization in PDAC, results in a much more severe hypoxia environment.^[^ ^6^ ^]^ CD73, a cell surface ecto‐5′‐nucleotidase coded by the gene NT5E, can be upregulated in response to low oxygen levels through HIF‐1α mediated mechanisms, then participating in the metabolism of extracellular ATP, which is a potent proinflammatory factor released upon cell death caused by hypoxia or chemotherapy.^[^ ^7^ ^]^ Specifically, in the presence of CD39, an ectonucleoside triphosphate diphosphohydrolase‐1 coded by the gene ENTPD1, ATP is hydrolyzed to AMP. Subsequently, CD73 dephosphorylates AMP to adenosine. The accumulation of adenosine in the tumor microenvironment (TME) consequently impedes the infiltration of immune cells and dampens their functions.^[^ ^8^ ^]^ Commonly, adenosine exerts inhibitory effects on the activation and proliferation of T cells by binding to the A2A receptor on effector T cells.^[^ ^9^ ^]^ Furthermore, activation of adenosine signaling has been demonstrated to reduce the recruitment of neutrophils, either directly or indirectly, thereby inhibiting inflammation.^[^ ^10^ ^]^ Collectively, these findings provide compelling evidence for further investigation of targeting CD73 as a new exciting and promising cancer immunotherapy.

The correlation between elevated CD73 expression and unfavorable prognosis has been established in several cancers, including PDAC.^[^ ^11^ ^]^ Previously, we observed up‐regulation of CD73 in PDAC tissues, and we also observed a significant association between higher CD73 expression, increased programmed death‐1 receptor (PD‐L1) expression, and poorer prognosis.^[^ ^11d^ ^]^ These findings underscore the potential of CD73 as a promising target to facilitate the efficacy of immunotherapy. So far, multiple small molecule inhibitors or monoclonal antibodies (mAbs) targeting CD73 have gradually emerged for cancer treatment.^[^ ^8^ ^]^ Small molecule inhibitors might be more efficient for tissue penetration and tumor retention than mAbs, primarily due to their small molecular weight.^[^ ^12^ ^]^ This is particularly relevant in the context of pancreatic cancer, where the microenvironment is characterized by dense desmoplasia. As one of the first small inhibitors of CD73, adenosine 5′‐(α,β‐methylene) diphosphate, has been extensively utilized in preclinical studies focusing on tumors.^[^ ^13^ ^]^ However, the subsequent extensive interrogation of structure‐based drug design, and optimization of pharmacokinetic properties culminated in the discovery of AB680, a highly potent (Ki = 5 pM), reversible, and selective inhibitor of CD73, which is further characterized by better tolerance, lower clearance, and longer half‐lives.^[^ ^14^ ^]^ Currently, only AB680 has been evaluated in the early phase of clinical trials against advanced pancreatic cancer. Preliminary data from the phase I clinical trials (NCT04104672) indicate that AB680, when administered in combination with chemotherapy and anti‐programmed death‐1 receptor (PD‐1) administration, revealed a manageable safety profile, accompanied with an overall response rate of 41%.^[^ ^15^ ^]^ Despite these findings, the detailed efficacy and mechanism of AB680 in the treatment of PDAC remain unclear. Additionally, the effective combination of therapeutic strategies based on variations in the tumor microenvironment induced by AB680 also needs further exploration.

Here, we further validated the differential expression of CD73 at the protein level and its prognostic value in PDAC, as well as its association with tumor‐infiltrating CD8^+^ T cells. The overexpression of CD73 in cancer cells promoted tumor growth and decreased CD8^+^ T cell infiltration in immune‐competent mice, suggesting that targeting CD73 might be a promising therapeutic strategy for PDAC. AB680, a potent and selective inhibitor of CD73, was found to exert an anti‐tumor effect depending on the increased infiltration of responsive CD8^+^ T cells. In addition, the infiltration of myeloid‐derived suppressor cells (MDSCs) is one of the key factors to protect tumors from the immune system and immunotherapy.^[^ ^16^ ^]^ It was also increased by the upregulation of the chemokine CXCL5 in cancer cells, which is a well‐known ligand for CXCR2 on MDSCs. Notably, the combination of AB680, anti‐PD‐1 antibody, and CXCR2 inhibitor showed superior synergistic tumor suppression in vivo, providing an efficient and promising therapeutic strategy for PDAC.

## 2 Results

### 2.1 CD73 is Highly‐Expressed in Human PDAC Cells and Correlated with Shorter Survival

**Figure 1:**
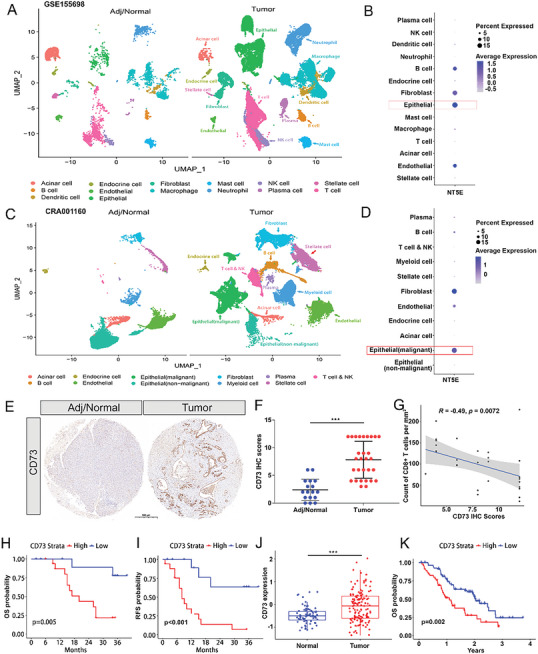
Human CD73 levels are upregulated in pancreatic cancer cells and correlate with survival. A) The distribution of defined cell clusters comparing three human adjacent/normal pancreas and 16 treatment‐naive PDAC tumors at the time of sample acquisition was shown by UMAP plots in the GSE155698 dataset. B) Dot plot of NT5E expression, the gene encoding CD73, in all identified cell clusters in the GSE155698 dataset C) the distribution of defined cell clusters comparing 11 human adjacent/normal pancreas and 24 treatment‐naive PDAC tumors at the time of sample acquisition was shown by UMAP plots in the CRA001160 dataset. D) Dot plot of NT5E expression, the gene encoding CD73, in all identified cell clusters in the CRA001160 dataset. E) Representative images of IHC staining of CD73 in human adjacent/normal pancreas and PDAC tumors. Scale bar: 500 µm. F) Quantification and statistical analysis of the above IHC staining of CD73 in 17 human adjacent/normal pancreas and 29 PDAC tumors without any treatment. CD73 IHC scores were compared between the two groups using the Mann–Whitney test. ^***^ *p* < 0.001 G) Correlation analysis of tumor‐infiltrating CD8^+^ T cell and CD73 IHC scores via Spearman correlation coefficients. H,I) Kaplan–Meier curves showing overall survival (H) and recurrence‐free survival (I) of patients with 29 PDAC stratified by CD73 IHC scores. *p*‐values are from log‐rank tests. J) Comparison of CD73 protein levels between 75 normal pancreas and 140 treatment‐naive PDAC tumors in the CTPAC dataset via Student's *t*‐test. ^***^ *p* < 0.001. Data presented as mean ± SD. K) Kaplan–Meier analysis for overall survival in 140 PDAC patients according to CD73 protein level via the log‐rank test.

Our previous study found that CD73 mRNA was upregulated in PDAC tissues, and associated with a poor prognosis and reduced CD8^+^ T cell infiltration, as analyzed through several publicly accessible databases.^[^ ^11d^ ^]^ To further investigate the importance of CD73 in PDAC, we sought to explore CD73 expression in detail by interrogating two independent single‐cell RNA (scRNA) sequencing datasets. These datasets showed that CD73 was predominantly expressed in malignant epithelial cells, fibroblasts, endothelial cells, and B cells in both datasets (**Figure** **1**A–D). We then utilized immunofluorescence staining to further confirm the co‐localization of CD73 and CK‐19 in PDAC cells (Figure S1, Supporting Information), thus supporting the highly expressed CD73 in PDAC cells.

Next, we examined CD73 protein expression by immunohistochemistry (IHC) on a tissue microarray and found significantly elevated CD73 expression in PDAC tissues compared to their adjacent normal tissues (Figure 1E,F). CD73 has been shown to participate in the generation of immunosuppressive adenosine, inhibiting the activation and proliferation of T cells. Our previous study also found an association between intratumoral CD73 expression and presumed CD8^+^ T cell infiltration through bioinformatics analysis.^[^ ^8^, ^11^ ^]^ As expected, CD73 expression was negatively correlated with the infiltrating level of CD8^+^ T cells (Figure 1G), and patients with higher CD73 expression had significantly shorter overall survival (OS) and recurrence‐free survival (RFS) (*p* = 0.005 and p <0.001, respectively) (Figure 1H,I). Furthermore, we recruited another cohort from the CTPAC dataset, and further demonstrated elevated CD73 expression in PDAC tissues, along with its prognostic values (Figure 1J,K). Thus, all these findings support the notion that CD73 might participate in PDAC progression and immunoregulation.

### 2.2 Overexpression of CD73 in Murine PDAC Cells Fosters Tumor Progression and Immune Evasion

**Figure 2:**
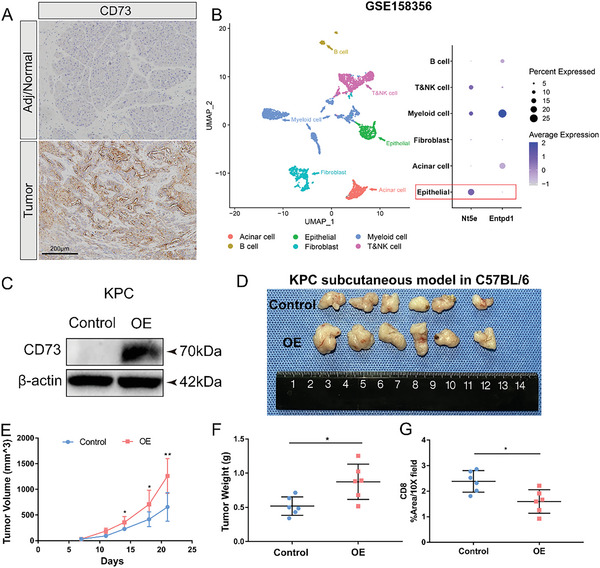
Overexpression of CD73 in murine tumor cells contributes to tumor growth in vivo. A) Representative images of immunohistochemistry staining of CD73 in murine adjacent/normal pancreas and orthotopic allografts of a KPC cell line. Scale bar: 200 µm. B) UMAP visualization of six identified cell populations and dot plot of Nt5e and Entpd1 expression in these populations in the single‐cell RNA‐sequencing dataset GSE158356 of two orthotopic allografts of a KPC cell line. C) Western blot analysis for CD73 in CD73 overexpressing (OE) and control KPC cells. D–F) Image (D), volumes (E), and weights (F) of the CD73 OE versus Control KPC tumors (*n* = 6 samples per group). Tumor volumes and weights were compared between Control and OE groups. Values are means ± SD, ^*^ *p* < 0.05, ^**^ *p* < 0.01 in a two‐sample *t*‐test. G) Quantification and comparison of immunohistochemistry result of CD8^+^ T cells in the CD73 OE versus Control KPC tumors (*n* = 6 samples per group). ^*^ *p* < 0.05, ^**^ *p* < 0.01 in Student's *t*‐test. Data presented as mean ± SD.

To further investigate the importance of tumoral CD73 in vivo, we established an orthotopic murine KPC model. Similarly, CD73 was found to be highly expressed in tumor epithelial cells compared to the adjacent normal pancreas (**Figure** **2**A). Analysis of a scRNA‐seq dataset for murine orthotopic allografts of a KPC cell line also revealed high and extensive expression of CD73 in tumor epithelial cells, while its partner involved in ATP metabolism, CD39, was highly expressed in myeloid cells (Figure 2B). We then constructed CD73‐overexpressing KPC cells and Panc02 cells using a lentiviral vector, as confirmed by the elevated expression of CD73 protein (Figure 2C; Figure S2A, Supporting Information). Overexpression of CD73 did not have an inhibitory effect on KPC or Panc02 cell proliferation in vitro (Figure S2B,C, Supporting Information), but it significantly increased tumor volume and weight compared to the control cells in both subcutaneously implanted murine models in C57BL/6 mice (Figure 2D–F; Figure S2D–F, Supporting Information). Furthermore, the numbers of total tumor‐infiltrating CD8^+^ T cells were significantly decreased in the CD73‐overexpressed KPC and Panc02 subcutaneous allografts compared with controls (Figure 2G; Figure S2G, Supporting Information). Additionally, the double immunofluorescence staining method was applied to evaluate the immunosuppressive role of CD73 on tumor‐infiltrating cytotoxic CD8^+^ T cells and found that the numbers of tumor‐infiltrating Granzyme B^+^CD8^+^ T cells were reduced in the CD73‐overexpressed KPC tumors compared to controls (Figure S2H, Supporting Information). These results suggested that CD73 may participate in PDAC progression in a microenvironment‐dependent manner and could be a potential therapeutic target for PDAC.

### 2.3 CD73 Inhibitor Abrogates the Tumor Burden and Prolongs Survival

**Figure 3:**
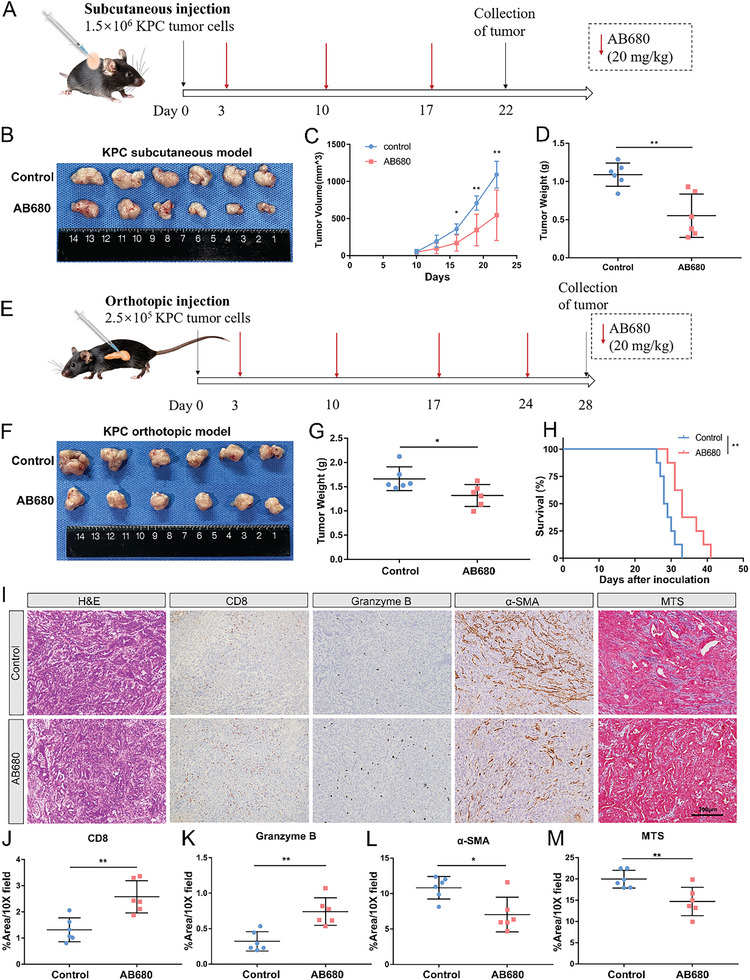
CD73 inhibition reduces the tumor burden and prolongs survival. A) Schematic showing the schedule of CD73 inhibitor AB680 treatment in KPC subcutaneous transplantation model. B–D) Image (B), volumes (C), and weights (D) of the AB680‐treated versus Control KPC subcutaneous allografts (*n* = 6 samples per group). Data presented as mean ± SD, ^*^ *p* < 0.05, ^**^ *p* < 0.01 in Student's *t*‐test. E) Schematic showing the schedule of AB680 treatment in KPC orthotopic transplantation model. F,G) Image (F) and weights (G) of the AB680‐treated versus Control KPC orthotopic allografts (*n* = 6 samples per group). Data presented as mean ± SD, ^*^p < 0.05 in Student's *t*‐test. H) Kaplan–Meier survival analysis of tumor‐bearing mice untreated or treated with 20 mg k^−1^g AB680 via the log‐rank test (*n* = 8 samples per group). I) Representative images of H&E staining, Masson's trichrome staining (MTS), and immunohistochemistry staining of CD8, Granzyme B, α‐SMA. Scale bar: 200 µm. J–M) Quantification of CD8 (J), Granzyme B (K), α‐SMA (L), and MTS (M) % positive area in the AB680‐treated versus Control KPC orthotopic allografts (*n* = 6 samples per group). Data presented as mean ± SD, ^*^ *p* < 0.05, ^**^ *p* < 0.01 in Student's *t*‐test.

AB680, a reversible and selective inhibitor of CD73, is currently is presently undergoing evaluation in early‐stage clinical trials owing to its remarkablely extended half‐lives and favorable tolerability.^[^ ^14^, ^15^ ^]^ In immune‐competent mice with KPC and Panc02 subcutaneously implanted tumor models, tumor volume and weight of AB680‐treated allografts were significantly reduced compared to controls (**Figure** **3**A–D; Figure S3A–D, Supporting Information). To better replicate the phenotypic and histological characteristics of pancreatic tumors, orthotopic transplantation models were established in immune‐competent mice. Consistently, the AB680 group exhibited significantly smaller tumors at the endpoint compared to the control group. Moreover, AB680 significantly prolonged survival in the orthotopic KPC transplantation tumor models (Figure 3E–H; median survival time: Control, 28.5 days; AB680, 33 days, p <0.01). Consistent results were observed in the orthotopic Panc02 transplantation tumor models (Figure S3E–H, Supporting Information). After AB680 treatment, the numbers of CD8^+^ and Granzyme B^+^ cells were significantly increased, as evidenced by IHC staining (Figure 3I–K). Dense desmoplastic stroma has been widely recognized as a typical feature of PDAC and contributes to progression and drug resistance. To explore the potential impact of inhibiting adenosine generation on the architecture of tumor‐surrounding stroma, trichrome staining and activated fibroblast marker (α‐SMA) staining were also applied. Our findings revealed that AB680 treatment reduced the infiltrating number of activated fibroblasts and collagen deposition (Figure 3L,M). However, the in vivo efficacy of AB680 was in suppressing tumor growth not observed in nude mice (Figure S4A–D, Supporting Information). Similarly, the in vitro experiments evaluating the effects of AB680 on the proliferation and clone formation ability of murine and human pancreatic cancer cell lines did not yield significant results (Figure S4E–H, Supporting Information), suggesting that the antitumor response of AB680 was dependent on the PDAC microenvironment, rather than directly inhibiting tumor cells.

### 2.4 AB680 Reprograms the Tumor Microenvironment and Exerts Antitumor Response Dependent on CD8^+^ T Cells

**Figure 4:**
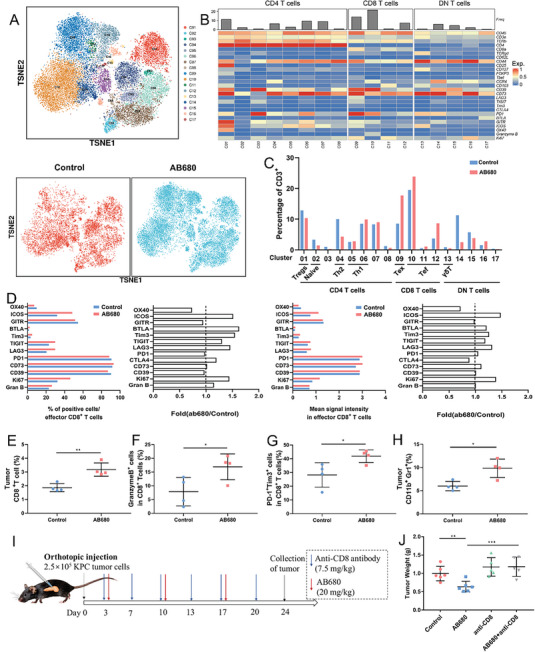
AB680 reprograms the tumor microenvironment and exerts an antitumor response dependent on CD8^+^ T cells. A) The t‐distributed stochastic neighbor embedding (t‐SNE) plot of CD3^+^ tumor‐infiltrating lymphocytes of the total six samples, control and AB680‐treated samples. B) Heatmap indicating the expression of markers specific for identifying differentiation, activation, and exhaustion status of T cells in 17 clusters. C) The frequencies of each cell cluster among the CD3^+^ cells in the two groups. D) The expression of immune checkpoints, Ki‐67, and Granzyme B in the tumor‐infiltrating CD8^+^ T cells from control mice or mice treated with AB680. E–H) The percentages of CD8^+^ T cells (E), Granzyme B^+^ (F), and PD1^+^TIM3^+^ cells (G) among the total CD8^+^ T cells, and MDSCs (H) in control and AB680‐treated KPC orthotopic allografts determined by flow cytometry (*n* = 4 samples per group). Data presented as mean ± SD, ^*^p < 0.05, ^**^p < 0.01 in Student's *t*‐test. I) Schematic representation of the therapy schedule for AB680 and anti‐CD8 antibody in the KPC orthotopic model. J) The weight of the orthotopic KPC allografts from the Control (*n* = 6), AB680(*n* = 6), anti‐CD8(*n* = 5), and AB680+anti‐CD8(*n* = 5) groups. ^**^ *p* < 0.01, ^***^ *p* < 0.001 in Student's *t*‐test. Data presented as mean ± SD.

To evaluate the overall immunological changes induced by inhibiting CD73 activity within tumors, we conducted a profiling analysis of tumor‐infiltrating immune cells (TILs) in the AB680‐treated and control KPC orthotopic allografts from immune‐competent mice using mass cytometry (CyTOF). tSNE plots and heatmaps were applied to illustrate alterations in the CD45^+^ immune cell population, which identified 30 distinct clusters based on 42 markers (Figure S5A,B, Supporting Information). As expected, the administration of AB680 led to a noticeable expansion of the specific CD8 T‐cell subset, namely cluster 6. Additionally, it was observed that AB680 increased the infiltration of specific MDSC subsets, namely clusters 11, 14, and 17 (Figure S5C, Supporting Information). To gain a more comprehensive understanding of the changes in TILs after AB680 treatment, the analysis of the CD3^+^ T cell population revealed an obvious increase in the frequencies of exhausted CD8^+^ T cells (cluster 9) and effector CD8^+^ T cells (clusters 10–12), and a reduction in Th2 CD4^+^ T cell frequencies (cluster 4) in AB680‐treated tumors (**Figure** **4**A–C). Considering the vital role of CD8^+^ T cells in anti‐tumor immunity, the protein levels of multiple costimulatory, co‐inhibitory, cytotoxic, and proliferative molecules within CD8^+^ T cells were subsequently investigated. AB680 upregulated costimulatory and activated molecules such as inducible T‐cell costimulatory, granzyme B, and Ki‐67, and it also increased the expression of inhibitory molecules, including BLTA, TIM3, TIGIT, and LAG3 (Figure 4D). To validate the findings obtained from CyTOF analysis, conventional flow cytometry was employed and further confirmed that AB680 significantly increased the infiltration of responsive CD8^+^ T cells as well as MDSCs (Figure 4E–H), indicating that AB680 remodeled the tumor microenvironment by increasing the infiltration of both immune killer cells and immunosuppressive cells.

Given the significantly increased number and activity of CD8^+^ T cells in the orthotopic KPC model of AB680‐treated mice, accompanied by a reduction in tumor burden, we further investigated whether the antitumor effect of AB680 could be impaired by the depletion of CD8^+^ T cells (Figure 4I). Anti‐CD8 monoclonal antibody was applied to block CD8^+^ T cells in vivo, which was validated by flow cytometry (Figure S6A, Supporting Information). The antitumor effect of AB680 was reversed by CD8 depletion (Figure 4J; Figure S6B, Supporting Information). In addition, the significant expansion of CD8^+^ T cells observed in AB680‐treated tumors was effectively counteracted by the depletion effect of the anti‐CD8 antibody, as validated by IHC staining for CD8 (Figure S6C,D, Supporting Information). In brief, it could be inferred that CD8^+^ T cells are the key mediators for the antitumor response of AB680.

### 2.5 Combination of AB680 and Gemcitabine cannot Enhance the Effect to Suppress Tumor Growth

MDSCs have been recognized as significant contributors to immune evasion in various cancers.^[^ ^17^ ^]^ Gemcitabine, a widely used first‐line chemotherapeutic agent, has demonstrated efficacy in eliminating MDSCs at a low dose.^[^ ^18^ ^]^ Given the profound effect of the increased infiltration of MDSCs following AB680 treatment, which might partially compromise the antitumor efficacy, we hypothesized that gemcitabine could potentially augment the sensitivity to AB680. Consequently, we administered a combined treatment of AB680 and a low‐dose gemcitabine, or a single treatment to the tumor (Figure S7A, Supporting Information). Surprisingly, we observed no significant differences in efficacy among the AB680 group, gemcitabine group, and AB680+gemcitabine group. Nevertheless, all treatment groups significantly reduced the tumor volume and weight compared to the control group (Figure S7B,C, Supporting Information). Besides, low‐dose gemcitabine intervention could slightly reduce the weight of mice while AB680 treatment had no significant effect (Figure S7D, Supporting Information). To investigate the underlying mechanism behind the limited efficacy of AB680+gemcitabine therapy in augmenting the antitumor effect compared to AB680 or gemcitabine alone, we performed flow cytometry to analyze the changes in the tumor microenvironment following different treatments. Notably, gemcitabine alone and AB680+gemcitabine could significantly reduce the infiltration of MDSCs in the tumors; Besides, they also significantly decreased the fraction of overall tumor‐infiltrating CD8^+^ T cells (Figure S7E–H, Supporting Information). In line with these findings, IHC analysis of the tumor revealed that AB680+gemcitabine significantly decreased both the tumor‐infiltrating CD8^+^ T cells and MDSCs; In addition, no significant difference in the tumor‐infiltrating granzyme B^+^ cells among the treatment groups was identified (Figure S7I, Supporting Information). However, the antitumor effect of AB680 was dependent on the presence of CD8^+^ T cells as demonstrated above. Taken together, these observations collectively indicate that gemcitabine has the potential to compromise the efficacy of AB680 by reducing CD8^+^ T cells. Therefore, the combination of AB680 and gemcitabine might not be a viable treatment for PDAC.

### 2.6 AB680 Enhances the Sensitivity to PD‐1 Blockade in Orthotopic KPC Tumors

**Figure 5:**
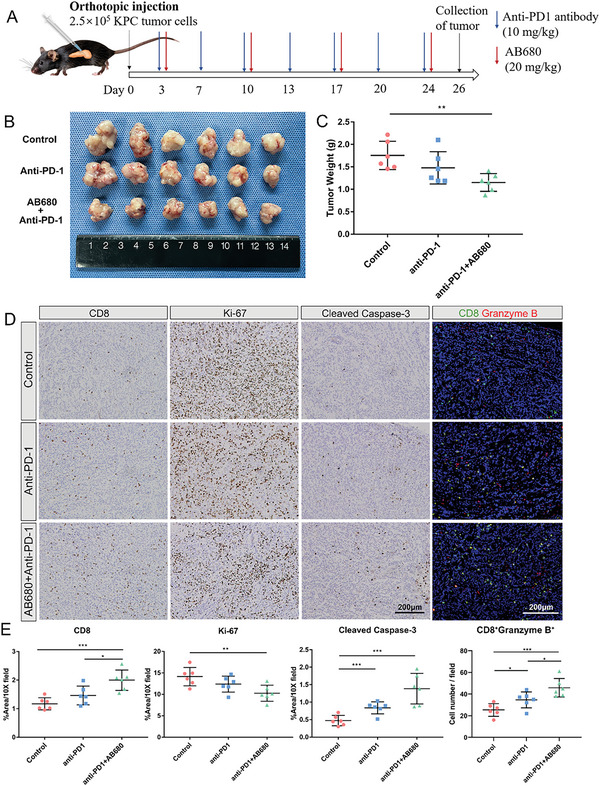
AB680 enhances the sensitivity to PD‐1 blockade for the treatment of orthotopic KPC allografts. A) Schematic representation of the therapy schedule for AB680 and anti‐PD‐1 in the KPC orthotopic model. B,C) Image (B) and tumor weight (C) of KPC orthotopic allografts from the Control, Anti‐PD‐1, and AB680+Anti‐PD‐1 groups (*n* = 6 samples per group). Data are presented as mean ± SD, with ^*^ *p* < 0.05 and ^**^ *p* < 0.01 in Student's *t*‐test. D) Representative images of immunohistochemistry staining of CD8, Ki‐67, and Cleaved caspase‐3, and immunofluorescence staining of Granzyme B^+^CD8 T cells in the KPC allografts from the indicated treatment groups (*n* = 6 samples per group). Scale bar: 200 µm. E) Quantification of CD8, Ki‐67, and Cleaved caspase‐3% positive area, and Granzyme B^+^CD8^+^ cell number in the tumors of each group at the endpoint. *n* = 6 samples per group. Data are presented as mean ± SD, with ^*^ *p* < 0.05, ^**^ *p* < 0.01, and ^***^ *p* < 0.001 in Student's *t*‐test.

There is a growing awareness that the lack of preexisting responsive T cells in PDAC might be a potential factor in inducing resistance to anti‐PD‐1 antibodies.^[^ ^19^ ^]^ These antibodies have previously been found to be ineffective in the KPC orthotopic model when used as a single agent.^[^ ^20^ ^]^ Our data demonstrated that the antitumor effect of AB680 depended on the expansion of responsive CD8^+^ T cells. A previous study also demonstrated the synergistic anti‐tumor effect of targeting CD73 and PD‐1 in mouse models of colorectal cancer.^[^ ^21^ ^]^ Therefore, we further explored whether inhibiting CD73 activity with AB680 to increase tumor‐infiltrating CD8^+^ T cells could improve the efficacy of anti‐PD‐1 treatment in PDAC (**Figure** **5**A). The treatment of immunocompetent mice harboring orthotopic KPC tumors with AB680+anti‐PD‐1 therapy resulted in a more remarkable inhibitory effect on tumor growth compared to control and PD‐1 blockade alone (Figure 5B,C). Besides, the numbers of total CD8^+^ T cells, granzyme B^+^ CD8^+^ T cells, and apoptotic cells, as measured by cleaved caspase‐3 staining, were significantly increased. Ki‐67 staining revealed a significant decrease in proliferative cells within tumors after AB680+anti‐PD‐1 therapy (Figure 5D,E). These findings suggest that targeting CD73 might provide an opportunity for immunotherapy in PDAC, as we previously reported.

### 2.7 AB680 Activates CXCL5 Expression in Tumor Cells to Recruit MDSCs

**Figure 6:**
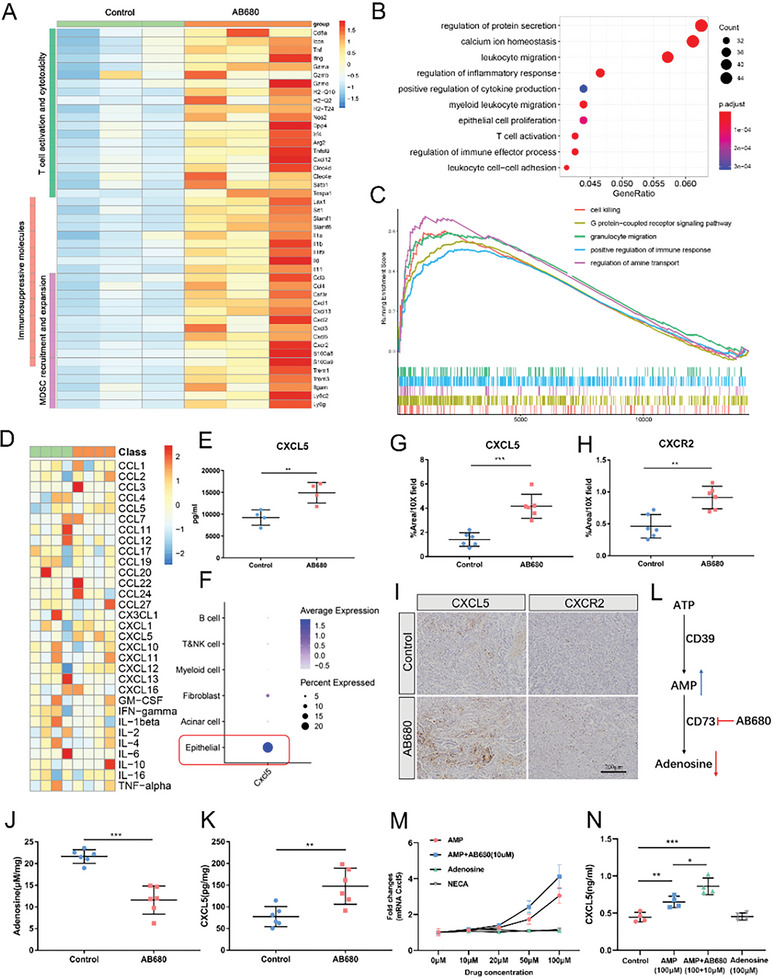
AB680 regulates CXCL5 released by tumor cells and promotes MDSC accumulation. A) Heatmap of immune‐related differential genes in the RNA‐seq data of control and AB680‐treated tumors (*n* = 3 samples per group). B,C) Over‐Representation Analysis B) and Gene Set Enrichment Analysis (C) of the above RNA‐seq data. D) Heatmap of 23 different mouse cytokine expression levels in serums obtained at the time of tumor collection, as shown in Figure 3E using Bio‐Plex Pro Mouse Cytokine 23‐plex immunoassay (*n* = 4 samples per group). E) The protein level of CXCL5 in mouse serums from the indicated treatment groups. Data presented as mean ± SD, ^**^ *p* < 0.01 in Student's *t*‐test. F) Dot plot of Cxcl5 expression in the identified populations in the single‐cell RNA‐sequencing dataset GSE158356 of two orthotopic KPC allografts. G,H) Quantification of CXCL5 and CXCR2% positive area in the AB680‐treated versus Control KPC tumor at the endpoint (*n* = 6 samples per group). ^**^ *p* < 0.01, ^***^ *p* < 0.001 in the Student's *t*‐test. Data presented as mean ± SD. I) Representative images of immunohistochemistry staining of CXCL5 and CXCR2 in the KPC tumors from the indicated treatment groups. J,K) The level of adenosine and CXCL5 protein in the AB680‐treated versus Control KPC tumor at the endpoint (*n* = 6 samples per group). ^**^ *p* < 0.01, ^***^ *p* < 0.001 in Student's *t*‐test. Data presented as mean ± SD. L) Schematic representation of the changes in adenosine metabolism after inhibition of CD73 activity by AB680. M) qRT‐PCR analysis of Cxcl5 mRNA expression in the KPC cells with different concentrations of AMP, adenosine, and the adenosine receptor agonist NECA at 0, 10, 20, 50, and 100 µm (*n* = 3). Data presented as mean ± SD. N) The CXCL5 protein concentration in the culture supernatant was evaluated by ELISA in the KPC cells with indicated concentrations of drugs (*n* = 3). Data presented as mean ± SD. ^*^ *p* < 0.05, ^**^ *p* < 0.01, ^***^ *p* < 0.001 in Student's *t*‐test.

To dissect the mechanism by which AB680 promotes MDSC accumulation, RNA sequencing was conducted in control and AB680‐treated tumors. Compared to control tumors, a total of 525 genes were upregulated, while 268 genes were downregulated in AB680‐treated tumors. Notably, T‐cell activated and cytotoxic markers, such as Tnf, Ifng, and Gzmb, as well as genes with immunosuppressive and MDSC recruitment function including Cxcl5 were significantly upregulated in AB680‐treated tumors (**Figure** **6**A). GO enrichment analysis revealed that the biological processes in which these differential genes participated were enriched in the regulation of cytokine production and secretion, T‐cell activation, and myeloid leukocyte migration (Figure 6B). The GSEA analysis also demonstrated consistent findings (Figure 6C), providing additional evidence to the fact that the MDSC accumulation following AB680 treatment might be attributed to chemotaxis. Therefore, we next collected the serum for Cytokine 23‐plex immunoassay (Figure 6D). Remarkably, the serum level of CXCL5 was significantly upregulated in mice treated with AB680 compared to the control mice (Figure 6E). CXCL5, known as a ligand for CXCR2, has been widely reported as an effective chemoattractant for MDSCs.^[^ ^22^ ^]^ Further analysis of scRNA‐seq data for orthotopic pancreatic tumors of mice unveiled that cancer epithelial cells predominantly served as the primary producers of CXCL5 (Figure 6F). Moreover, IHC staining for CXCL5 in the KPC orthotopic tumors further confirmed that CXCL5 expression was predominantly localized in malignant epithelial cells. Notably, the percentage of CXCL5^+^ area was observed to be significantly increased in AB680‐treated tumors (Figure 6G,I). The increased percentage of corresponding receptor CXCR2^+^ area was also confirmed by immunohistochemistry in AB680‐treated tumors (Figure 6H,I).

Next, we further investigated the mechanism underlying the increased secretion of CXCL5 on cancer cells following AB680. As expected, adenosine levels were significantly decreased in AB680‐treated tumors compared to control tumors due to the enzyme activity of CD73 being inhibited by this small‐molecule inhibitor (Figure 6J). Additionally, the upregulation of the CXCL5 protein was further validated in AB680‐treated tumors in vivo via ELISA (Figure 6K). Interestingly, it was observed that AB680 had no direct effects on the expression of CXCL5 compared to the untreated cells in vitro (Figure S8A, Supporting Information). The substrate AMP for CD73 was the main downstream product of ATP metabolism, which was catabolized by CD39 (Figure 6L). Notably, CD39 was highly expressed in myeloid cells, rather than in cancer cells (Figure 2B). Considering the extremely low extracellular AMP levels observed in vitro cancer cell culture processes, attributed to the lack of CD39, we introduced the AMP as an approach to mimic the metabolic environment following AB680 treatment in vivo (Figure 6L). The Cxcl5 mRNA level was significantly upregulated by AMP in a dose‐dependent manner. However, neither the product adenosine nor the adenosine receptor agonist demonstrated a similar effect. Furthermore, the Cxcl5 expression was higher in the AMP+AB680 group compared to the AMP group (Figure 6M). ELISA was applied to compare the CXCL5 protein concentration in the culture supernatant and found consistent outcomes (Figure 6N). Additionally, the higher expression of the human homolog of mouse Cxcl5, CXCL6, and other ligands of CXCR2 (CXCL1 and CXCL2) was also identified in human pancreatic cancer cells after adding AMP. However, this effect was not observed when adenosine was added (Figure S8B, Supporting Information). These findings potentially suggest that AB680 could upregulate AMP levels to enhance the recruitment of CXCR2^+^ MDSCs.

### 2.8 CXCR2 Inhibitor Further Potentiates the Efficacy of AB680 Plus Anti‐PD‐1 Therapy

**Figure 7:**
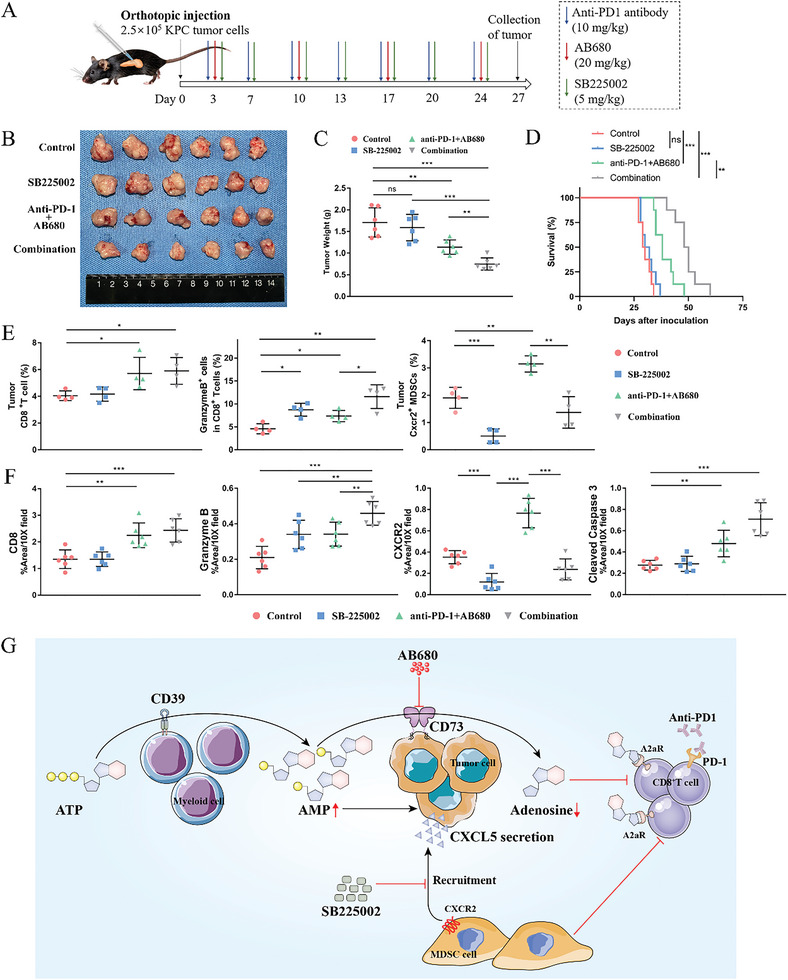
CXCR2 inhibition further potentiates the efficacy of AB680 plus anti‐PD‐1 therapy. A) Schematic representation of the therapy schedule for AB680, anti‐PD‐1 antibody, and CXCR2 inhibitor SB225002 in the KPC orthotopic model. B,C) The image (B) and tumor weight (C) of KPC orthotopic allografts from the Control, SB225002, anti‐PD‐1+AB680, and combination groups (*n* = 6/group). ^**^ *p* < 0.01, ^***^ *p* < 0.001 in Student's *t*‐test. Data presented as mean ± SD. D) Survival of immunocompetent mice harboring KPC orthotopic allografts treated with the indicated treatment compared with the survival of those untreated mice (*n* = 8/group). **p < 0.01, ^***^ *p* < 0.001 in the log‐rank test. E) The percentages of total CD8^+^ T cells, Granzyme B+ cells among the total CD8^+^ T cells, and CXCR2^+^ MDSCs in the tumors of each group at the endpoint were determined by flow cytometry. *n* = 4 samples per group. Data presented as mean ± SD. ^*^ *p* < 0.05, ^**^ *p* < 0.01, ^***^ *p* < 0.001 in Student's *t*‐test. F) Quantification of CD8, Granzyme B, CXCR2, and Cleaved caspase‐3% positive area in the tumors of each group at the endpoint. *n* = 6 samples per group. Data presented as mean ± SD. ^**^ *p* < 0.01, ^***^ *p* < 0.001 in Student's *t*‐test (G) schematic diagram depicting the mechanism by which CXCR2 inhibitor further potentiates the efficacy of AB680 plus anti‐PD‐1 therapy. AB680 can significantly increase the infiltration of responsive CD8+ T cells by suppressing the production of immunosuppressive adenosine, but it also promotes the MDSC chemotaxis by tumor‐derived CXCL5 in an AMP manner. Targeting the CXCL5/CXCR2 pathway with the CXCR2 inhibitor SB‐225002 can disrupt the “side effect” of AB680 and potentiate the efficacy of AB680 plus anti‐PD‐1 therapy. Data presented as mean ± SD, Student's *t*‐test was used unless otherwise indicated.

The above findings inspired us to explore whether disrupting the chemotaxis of MDSCs by a CXCR2 inhibitor could improve the therapeutic efficacy of AB680+anti‐PD‐1 therapy. Thus, we constructed the orthotopic KPC model and administered the CXCR2 inhibitor (SB225002), AB680+anti‐PD‐1 therapy, or a combination of those drugs 3 days after tumor transplantation (**Figure** **7**A). The combination therapy administered to immunocompetent mice harboring KPC tumors triggered a more distinct suppressive effect on tumor growth and resulted in prolonged survival compared to the control group or either therapy administered alone (Figure 7B–D; median survival time: Control, 29.5 days; SB‐225002, 31 days; anti‐PD‐1+AB680, 38 days; Combination, 49 days), while no significant difference in weight was identified between the treatment groups at the endpoint (Figure S9A, Supporting Information). In addition, flow cytometry analysis revealed that mice treated with the combination therapy exhibited more cytotoxic CD8^+^ T cells, while the increased infiltration of MDSCs induced by AB680+anti‐PD‐1 therapy was reversed (Figure 7E). Then, we further performed IHC staining and found consistent results (Figure 7F; Figure S9B, Supporting Information), which supported that the concurrent administration of a CXCR2 inhibitor targeting the CXCL5 cognate receptor, AB680, and PD‐1 inhibitor has the potential to impede the recruitment of immunosuppressive MDSCs caused by AB680 and significantly boost the antitumor immune responses to restrain tumor growth.

## 3 Discussion

In this study, we found that AB680 could significantly reduce the tumor burden and improve survival in immune‐competent mice. Both tumor‐infiltrating cytotoxic and exhausted CD8^+^ T cells were significantly increased in AB680‐treated tumors, which was consistent with prior publications demonstrating its ability to enhance the proliferation and activation of CD8^+^ T cells in vitro.^[^ ^21^ ^]^ Furthermore, CD73 overexpression in cancer cells has been reported to eliminate the favorable prognosis associated with high tumor‐infiltrating CD8^+^ T cells in patients with high‐grade serous ovarian cancer.^[^ ^11c^ ^]^ However, our data demonstrated that the efficacy of targeting CD73 by AB680 was abolished in lymphocyte‐deficient and CD8‐deficient tumor‐bearing mice. These results further support that the antitumor effect of AB680 depends on increased infiltration of CD8^+^ T cells. PD‐1 is an inhibitory checkpoint receptor that interacts with PD‐L1 to induce immune evasion. Blocking the PD‐L1/PD1 axis has been shown to be an effective therapy for targeting tumors with an inflamed tumor microenvironment, including melanoma, bladder cancer, gastric cancer, and non‐small cell lung cancer.^[^ ^23^ ^]^ However, PDAC is generally considered a non‐immunogenic tumor with limited infiltration of activated T cells. Numerous studies have shown that the presence of tumor‐infiltrating T cells is necessary for an acceptable response to immune checkpoint inhibition. Increasing T‐cell infiltration into PDAC might contribute to improving the clinical responses to PD‐1 blockade.^[^ ^24^ ^]^ Therefore, we conducted a combination therapy of AB680 and anti‐PD‐1, which demonstrated superior synergistic tumor suppression in vivo, consistent with previous studies showing that targeting CD73 could enhance the antitumor activity of immune checkpoint blockade.^[^ ^25^ ^]^

In addition to increasing responsive tumor‐infiltrating CD8^+^ T cells, AB680 also increased the infiltration of MDSCs. MDSCs have been shown to promote cancer progression and metastasis through various mechanisms, such as angiogenesis, epithelial‐mesenchymal transition, and the release of tumor‐promoting molecules. They also induce the immune effector cell anergy mainly by upregulating the enzymes IDO, arginase‐1(ARG1), and inducible nitric oxide synthase (iNOS).^[^ ^16^ ^]^ This might explain the simultaneous elevation of exhausted CD8^+^ T cells following AB680 treatment in our work. Inhibition of T‐cell responsiveness by MDSCs has been shown to be correlated with poor efficacy of immunotherapies.^[^ ^26^ ^]^ Therefore, we investigated the ways to counteract MDSCs to restore the antitumor activities of immune effector cells and promote synergistic tumor suppression with AB680 and PD‐1 blockade. Coincidentally, gemcitabine, the first‐line chemotherapy for PDAC, has been reported to deplete tumor‐infiltrating MDSCs at low doses.^[^ ^18^, ^27^ ^]^ However, combining gemcitabine with AB680 did not result in a more significant decrease in tumor volumes compared to either treatment alone, as it weakened the efficacy of AB680 by reducing CD8^+^ T cells. Although CD73 has been reported to promote gemcitabine resistance in pancreatic cancer cells, its role in gemcitabine resistance is mediated by its ecto‐5′‐nucleotidase‐independent functions by regulating CD73 expression through knockdown and overexpression.^[^ ^28^ ^]^ However, AB680 could not regulate the expression of CD73 in tumor cells, and exert anti‐tumor effects by inhibiting its enzyme activity.

The failure of gemcitabine combined with AB680 led us to target the factors contributing to increased MDSC infiltration. Through RNA sequencing, we found that pathways involved in the regulation of cytokine production and secretion, as well as myeloid leukocyte migration, were significantly enriched in AB680‐treated tumors. The recruitment of MDSCs to tumors is regulated by multiple chemoattractants secreted by cancer cells and stroma cells, such as CCL1‐3, CXCL1, CXCL5, CXCL8, and S100A8/9.^[^ ^16^ ^]^ In this study, CXCL5 was identified to be significantly upregulated in the serum and tumor of mice following AB680 treatment, as confirmed by a 23‐cytokine immunoassay and ELISA. scRNA‐seq analysis of KPC tumors revealed high expression of CXCL5 in cancer cells, which was consistent with previous studies.^[^ ^29^ ^]^ CXCL5, as a cancer‐secreted chemokine, has been shown to attract CXCR2^+^ MDSCs and promote the progression of primary melanoma and prostate tumors.^[^ ^30^ ^]^ The tumor‐promoting role of the CXCR2‐expressing MDSCs has also been identified in genetically engineered mice that spontaneously develop aggressive tumors.^[^ ^31^ ^]^ Additionally, knockout of Cxcr2 in mice prolonged survival in a mouse model of PDAC via a shift in the immune‐inflammatory microenvironment.^[^ ^32^ ^]^ Our findings of AB680 upregulating tumor‐derived CXCL5 secretion suggest that blocking the chemokine receptor CXCR2 might sensitize PDAC to AB680 therapy. Targeting the chemokine receptor CXCR2 to prevent MDSC recruitment to the tumor microenvironment has been reported to suppress metastases, improve T cell entry, and augment the efficacy of PD‐1 blockade.^[^ ^22^ ^]^ This is consistent with our findings that the combination therapy of a CXCR2 inhibitor, AB680, and anti‐PD‐1 therapy showed superior synergistic tumor suppression and prolonged the survival of tumor‐bearing mice. Due to the significant protumor functions of CXCR2, CXCR2 inhibitors have been evaluated in many clinical trials for treating cancers, as summarized in recent reviews.^[^ ^33^ ^]^ The effectiveness of inhibiting CXCR2 in our model suggests that targeting the mechanism that facilitates MDSC recruitment would provide better therapeutic benefits for PDAC patients receiving AB680 treatment.

However, there are some limitations to this study. First, we did not reveal the mechanism by which the secretion of CXCL5 in cancer cells was upregulated by AB680 due to the lack of the substrate of CD73 enzyme in vitro, although we used an additional substrate in this study to mimic the increased AMP level in vivo. A previous study identified the specific binding of AMP to the adenosine A1 receptor (ADORA1),^[^ ^34^ ^]^ and ADORA1 was found to promote tumor progression via the PI3K/AKT signaling pathway.^[^ ^35^ ^]^ Activation of the AKT signaling pathway effectively promotes SOX9 expression,^[^ ^36^ ^]^ which has been reported to transcriptionally regulate CXCL5 expression.^[^ ^29^, ^37^ ^]^ Therefore, we speculated that AMP might upregulate CXCL5 expression through the ADORA1/AKT/SOX9 axis, which will be further verified in our subsequent study. Second, immune suppression may be mediated by monocytic and granulocytic MDSC populations, both of which express CXCR2 on their cell surfaces. Although the CXCL5‐CXCR2 axis is identified as an effective target regardless of the monocytic and/or granulocytic MDSC populations, we will further identify the specific populations that affect the efficacy of AB680 treatment in our subsequent study. Lastly, human and mouse MDSCs may differ to some extent even though both are heterogeneous populations of immature myeloid cells.^[^ ^38^ ^]^ Although the KPC orthotopic tumor transplantation model has been relatively good at mimicking human PDAC in preclinical studies, the findings in this study only provide hints for clinical combination therapy, and further exploration and verification are needed in future clinical practice.

In summary, our study reveals that CD73 on PDAC cells plays a role in cancer progression. Targeting its enzyme activity with AB680 significantly increases the infiltration of responsive CD8^+^ T cells and prolongs the survival of mice. However, it also promotes MDSC chemotaxis through tumor‐derived CXCL5 in an AMP‐dependent manner. Therefore, combining a CXCR2 inhibitor with AB680 and PD‐1 inhibitor presents a promising antitumor efficacy, providing a novel potential therapeutic strategy for PDAC.

## 4 Experimental Section

### Analysis of Single‐Cell RNA Sequencing (scRNA‐Seq) and Proteome Data

The two processed human scRNA‐seq datasets were available in the Genome Sequence Archive under project PRJCA001063 and the NIH GEO database (Accession #GSE155698).^[^ ^39^ ^]^ The processed scRNA‐seq datasets for the two mouse orthotopic allografts of a KPC cell line were obtained from GEO (Accession #GSE158356).^[^ ^40^ ^]^ The downstream analyses were conducted using Seurat V4.0.5. Graph‐based clustering of the principal components was applied to distinguish the cell types, which were annotated based on the common marker genes as described previously,^[^ ^39^ ^]^ and Dotplot was utilized to present the expression of specific markers in relevant cell subtypes.

The proteome dataset PDC000270^[^ ^41^ ^]^ obtained from CTPAC (https://pdc.cancer.gov/pdc/) included 75 normal‐appearing tissues and 140 PDAC samples with complete survival information. It was used to validate the differential expression and prognostic value of the CD73 protein in PDAC.

### IHC and Immunofluorescence

A tissue microarray of 31 resected PDAC tissues was prepared for staining as previously described.^[^ ^42^ ^]^ The design of this study was approved by the Ethics Committee of Zhongshan Hospital, Fudan University (No. B2020‐346R). Formalin‐fixed, paraffin‐embedded sections of humans and mice were processed and incubated with primary antibodies: CD73 (Proteintech, 12231‐1‐AP), CK19 (Proteintech, 10712‐1‐AP), α‐SMA (Boster, BM0002), CD8 (Abcam, ab209775), granzyme B (Abcam, ab4059), CXCR2 (Proteintech, 19538‐1‐AP), CXCL5 (R&D Systems, AF433), LY6G (Servicebio, GB11229), Ki‐67 (CST, 9129), and cleaved caspase 3 (CST, 9664), followed by biotinylated or fluorescent secondary antibodies. The nuclei were stained with hematoxylin or DAPI. Hematoxylin and eosin (H&E) and Masson's trichrome staining were conducted using the H&E Staining Kit (Baso, BA4041) and Masson's Trichrome Stain Kit (Biossci, BP0310), respectively, following the manufacturer's instructions, respectively. All steps of staining, imaging, and quantification were carried out blinded to the sample identity.

### Cell lines and Culture

The human‐derived PC cell lines PANC‐1 and BxPC‐3 were obtained from the Chinese Academy of Sciences. Mouse‐derived PC cell lines Panc02 cells and LSL‐K‐ras^G12D/+^; Trp53^R172H/+^; Pdx‐1^Cre/+^ (KPC) cells were kind gifts from Johns Hopkins Hospital. All cells were kept in an incubator at 37 °C with 5% CO2. PANC‐1, BxPC‐3, as well as KPC cells were maintained in high‐glucose DMEM (Gibco) with 10% FBS (Gibco). Panc02 cells were cultured in RPMI‐1640 medium (Gibco) supplemented with 10% FBS. The above cell lines were authenticated through STR and SNP profiling over the last 3 years.

### Cell Transfection

The mouse Nt5e (NM_011851) was cloned into the pLenti CMV empty vector. The lentivirus (Ubi‐Nt5e‐3FLAG‐SV40‐EGFP‐IRES‐puromycin) was constructed after the vectors were transfected into HEK293T cells and infected Panc02 or KPC cells. Then, the stably transfected cells were selected in the presence of 2 µg mL^−1^ puromycin (Gibco) and further confirmed by qRT‐PCR and western immunoblot.

### qRT‐PCR, RNA‐Seq, and Analysis of Differentially Expressed Genes

For qRT‐PCR, the procedures of RNA isolation, reverse transcription, and qRT‐PCR were performed as previously described.^[^ ^43^ ^]^ The sequences of primers used for real‐time qRT‐PCR were as follows: mouse Nt5e, 5’‐GCAGCATTCCTGAAGATGCG‐3’ (forward) and 5’‐CTCCCGAGTTCCTGGGTAGA‐3’ (reverse); mouse Cxcl5, 5’‐GCCCTACGGTGGAAGTCATA‐3’ (forward) and 5’‐GAGTGCATTCCGCTTAGCTT‐3’ (reverse); mouse Actb, 5’‐ACTGTCGAGTCGCGTCCA‐3’ (forward) and 5’‐TCATCCATGGCGAACTGGTG‐3’ (reverse); human CXCL1 5’‐TTCTGAGGAGCCTGCAACAT‐3’ (forward) and 5’‐CCCTTTGTTCTAAGCCAGAAACAC‐3’ (reverse); human CXCL2 5’‐GAGATCAATGTGACGGCAGG‐3’ (forward) and 5’‐CTGCTCTAACACAGAGGGAAACA‐3’ (reverse); human CXCL5 5’‐GTGCAATTAACAAAGCTACTGCAAG‐3’ (forward) and 5’‐GGCATCTAAAAAGCTCAGCAATG‐3’ (reverse); human CXCL6 5’‐TGCGTTGCACTTGTTTACGC‐3’ (forward) and 5’‐CTTCCCGTTCTTCAGGGAGG‐3’ (reverse); human CXCL7 5’‐TTGTAGGCAGCAACTCACCC‐3’ (forward) and 5’‐TGCAAGGCATGAAGTGGTCT‐3’ (reverse); human CXCL8 5’‐TCTGCAGCTCTGTGTGAAGG‐3’ (forward) and 5’‐TTCTCAGCCCTCTTCAAAAACT‐3’ (reverse); human ACTB 5’‐CTCGCCTTTGCCGATCC‐3’ (forward) and 5’‐ATCCTTCTGACCCATGCCC‐3’ (reverse).

For mRNA sequencing, the extraction and quantification of RNA were performed as in the qRT‐PCR. RNA integrity was assessed using the Agilent 2100 Bioanalyzer (Agilent Technologies. Then, libraries were constructed using the VAHTS Universal V6 RNA‐seq Library Prep Kit and sequenced on the Illumina NovaSeq 6000 platform according to the manufacturer's instructions. 150 bp paired‐end reads in FASTQ format were generated for each sample and processed using FASTP to obtain clean reads, which were then mapped to the Mus musculus UCSC reference genome mm10. The read counts of each gene were obtained using HTSeq‐count.

The DESeq2 package was applied to identify differentially expressed genes with an absolute log2(fold change) >1 and *p* < 0.01. Gene with <5 reads were filtered out. Then, the potential biological functions of these genes were evaluated using Over‐Representation Analysis (ORA), and Gene Set Enrichment Analysis (GSEA) was used to validate the differentially enriched pathways between the two groups of samples using the “clusterProfiler” package in R software.

### Western Blot

Cells were lysed in RIPA buffer (Beyotime) containing 1% Protease and Phosphatase Inhibitor Cocktail (Thermo Fisher Scientific Cat# 78446) on ice. The sample lysates were centrifuged at 12 000g for 15 min at 4 °C. Then, the quantified protein was loaded into the wells in a 10% SDS‐PAGE gel (Epizyme) and transferred to a polyvinylidene fluoride membrane (PVDF, Millipore). After blocking with 5% milk for an hour at room temperature, the PVDF membrane was incubated with the following antibodies: CD73 (CST, 13160) and β‐Actin (CST, 4970). Finally, the blots were visualized using enhanced chemiluminescence reagents with the ChemiScope 6000 (CLINX) after incubation with an HRP‐conjugated secondary antibody.

### Syngeneic Murine PDAC Models and Treatment Procedures

Female C57BL/6 mice and BALB/c nude mice, aged 6–8 weeks old, were purchased from Shanghai Jie Si Jie Laboratory Animal Co., Ltd. The mice were maintained under pathogen‐free conditions. All procedures were approved by the Institutional Animal Care and Use Committee at Zhongshan Hospital, Fudan University. For the subcutaneous model, 3 × 10^6^ or 1.5 × 10^6^ Panc02 and KPC cells suspended in PBS were subcutaneously injected into the back of each mouse with a total volume of 100 µL. The mice were then randomly divided into different groups. The CD73 inhibitor, AB680 (MedChemExpress, HY‐125286), was intraperitoneally injected at a concentration of 20 mg kg^−1^ every week. Tumor volume was assessed every 3 days by calipers. The volume was calculated using the formula 1/2AB^2^, where A represented the long tumor diameter and B represented the short tumor diameter. The data were presented as mean ± SD in mm^3^.

For the orthotopic transplantation model, 2.5 × 10^5^ KPC or 5 × 10^5^ Panc02 cells suspended in PBS were 1:1 (v/v) mixed with Matrigel and slowly injected into the tail of the pancreas after the mice were anesthetized with pentobarbital. The tumor‐bearing mice were then randomly divided into different treatment groups and treated with 20 mg kg^−1^ AB680 every week, 7.5 mg kg^−1^ anti‐CD8 antibody (BioXcell, BE0061), 20 mg kg^−1^ Gemcitabine (MedChemExpress, HY‐B0003), 10 mg kg^−1^ anti‐PD1 antibody (BioXcell, BE0146) and 5 mg kg^−1^ CXCR2 inhibitor SB225002 (MedChemExpress, HY‐16711) twice a week alone or in combination by intraperitoneal injection according to the experimental objective. Mice in the placebo groups received PBS, IgG2b (BioXcell, BE0090), or IgG2a (BioXcell, BE0146) intraperitoneally as controls at the indicated time points. Mice from the same batch were euthanized to collect tumor tissues and serum for further analyses.

For the survival study, tumor‐bearing mice were monitored without any further treatment until they succumbed spontaneously, or were terminated upon the appearance of severe premorbid symptoms requiring euthanasia such as severe cachexia, ascites, hypothermia, and tumor size exceeding 2 cm.

### Cytometry by Time‐Of‐Flight (CyTOF)

The harvested tumor tissues were minced and single‐cell suspensions were isolated using the Mouse Tumor Dissociation Kit (Miltenyi Biotec) following filtration through 70 µm cell strainers. After erythrocyte lysis using Lysing Buffer (BD Biosciences, 555899), the single‐cell suspensions were stained with Cell‐ID Cisplatin‐194Pt for 5 min and then blocked with Blocking Solution for 20 min on ice. Cells were washed and incubated with a total of 42 pre‐mixed metal‐conjugated antibodies (Table S1, Supporting Information) following the manufacturer's instructions. The samples were acquired using a Helios mass cytometer (Fluidigm) and analyzed by PLTTech Inc. (Hangzhou, China), as previously described.^[^ ^20^ ^]^

### Flow Cytometry

The tumor tissues harvested from the C57BL/6 mice were processed into single‐cell suspensions as described above for CyTOF. The fluorochrome‐conjugated antibodies against CD45 (clone 30‐F11), CD3e (145‐2C11), CD8 (53‐6.7), Granzyme B (QA16A02), PD‐1 (29F.1A12), Tim‐3 (RMT3‐23), CD11b (M1/70), Gr‐1 (RB6‐8C5), and CXCR2 (SA044G4) were purchased from BioLegend (San Diego, CA). The staining was performed using the same methods as CyTOF. All samples were detected using a FACS Aria III flow cytometer (BD Biosciences) and processed using FlowJo software.

### Proliferation Assay

The viability and proliferation of cancer cells were measured at 24, 48, and 72 h by the cell counting kit‐8 (CCK‐8, Dojindo Molecular Technologies) according to the manufacturer's instructions after the appropriate quantities of cells were seeded in the 96‐well plates. Additionally, colony formation assays were performed. The appropriate quantities of cells were seeded and treated with the corresponding concentrations of drugs in six‐well plates and cultured for 10–14 days. Then, all wells of the plates were washed, fixed, and stained with 0.1% crystal violet (Beyotime) as previously described.^[^ ^44^ ^]^ Finally, the number of cell colonies was counted using the Image J software.

### 23‐Cytokine Immunoassay and ELISA

Peripheral blood was collected from mice via the orbital sinus after anesthesia and centrifuged at 3000 g for 20 min at 4 °C to obtain serum. The analysis of 23 mouse cytokines was performed using the Bio‐Plex Pro Mouse Cytokine 23‐plex immunoassay (Bio‐Rad) according to the manufacturer's instructions. Furthermore, the protein levels of CXCL5 and adenosine in KPC tumors and cell culture medium were detected using a mouse CXCL5 ELISA Kit (Boster, EK0919) and Adenosine Assay (Cell Biolabs, MET‐5090).

### Statistical Analysis

All statistical analyses were performed by R software (version 4.2.2) and GraphPad Prism (version 7.00) in this study. All results were presented as mean ± SD unless otherwise indicated. Continuous variables were compared between groups using Student's *t*‐test or Mann–Whitney U test, as appropriate. The survival curves were compared using a log‐rank test through the “survdiff” function of the “survival” package in R. The correlation between variables was calculated using Spearman or Pearson correlation coefficients. A value of *p* < 0.05 was considered statistically significant.

## Conflict of Interest

The authors declare no conflict of interest.

## Author Contributions

Q.C., H.Y., J.H., and Y.X. contributed equally to this study. Q.C., N.P., and W.L. conceived and designed this study. Q.C., H.Y., J.H., and Y.X. performed the experiments. Q.C. and N.P. contributed to the writing of the original draft. Q.C. and H.Y. analyzed the data. W.W., H.X., L.Z., C.S., W.W., and L.L. collected the samples. J.Y. provided scientific and technical support. L.L., N.P., and W.L. revised and approved the paper for submission. L.L., N.P., and W.L. contributed to the funding acquisition. All authors revised, read, and approved the final manuscript.

## Supporting information

Supporting Information

## Data Availability

The two processed human single‐cell RNA sequencing (scRNA‐seq) data were available in the Genome Sequence Archive under project PRJCA001063 and NIH GEO database (Accession #GSE155698). The processed scRNA‐seq datasets for the mouse orthotopic KPC tumor and normal pancreas were obtained from GEO (Accession #GSE158356). The proteome dataset PDC00027022 was obtained from CTPAC (https://pdc.cancer.gov/pdc/). The data generated in this study are included in this published article and available upon reasonable request from the corresponding authors.
