# Supplementary material for: Tumor Microenvironment Responsive CD8+ T Cells and Myeloid‐Derived Suppressor Cells to Trigger CD73 Inhibitor AB680‐Based Synergistic Therapy for Pancreatic Cancer
Source: Adv Sci (Weinh). 2023 Oct 22;10(33):2302498. doi: 10.1002/advs.202302498 (PMC10667825; doi:10.1002/advs.202302498)
Supplement: Supplementary file 1 — Supporting Information [file ADVS-10-2302498-s001.pdf]

ADVANCED SCIENCE
Open Access

Supporting Information

for *Adv. Sci.*, DOI 10.1002/advs.202302498

Tumor Microenvironment Responsive CD8^{+} T Cells and Myeloid-Derived Suppressor Cells to Trigger CD73 Inhibitor AB680-Based Synergistic Therapy for Pancreatic Cancer

Qiangda Chen, Hanlin Yin, Junyi He, Yuqi Xie, Wenquan Wang, Huaxiang Xu, Lei Zhang, Chenye Shi, Jun Yu, Wenchuan Wu, Liang Liu, Ning Pu* and Wenhui Lou**

Supporting Information

Tumor microenvironment responsive CD8^{+} T cells and myeloid-derived suppressor cells to trigger CD73 inhibitor AB680-based synergistic therapy for pancreatic cancer

Qiangda Chen, Hanlin Yin, Junyi He, Yuqi Xie, Wenquan Wang, Huaxiang Xu, Lei Zhang, Chenye Shi, Jun Yu, Wenchuan Wu, Liang Liu, Ning Pu*, Wenhui Lou**

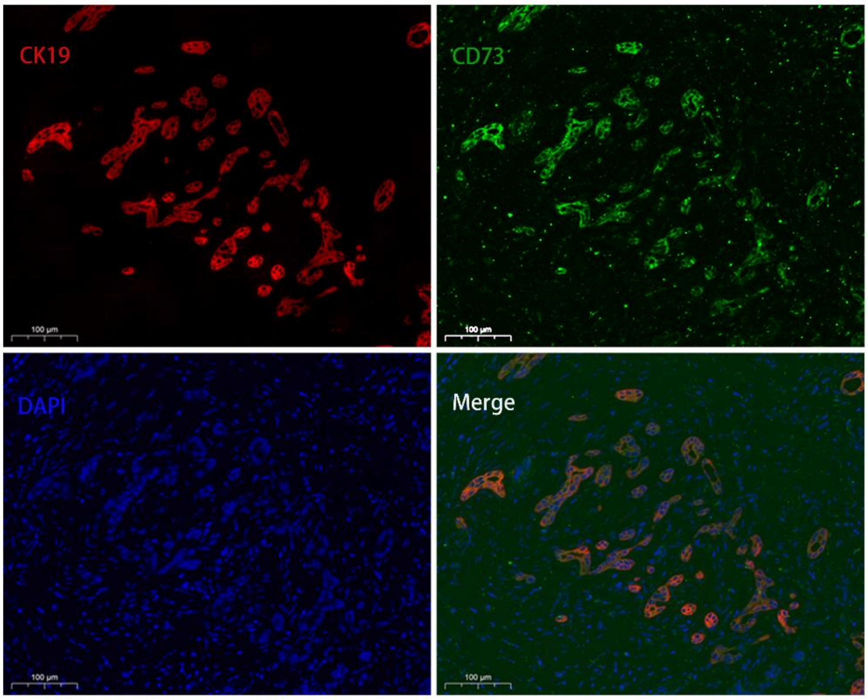

Supplementary Figure 1. Representative immunofluorescence staining of CD73 and CK19. CK19: red; CD73: green; DAPI: blue. Scale bar: 200μm.

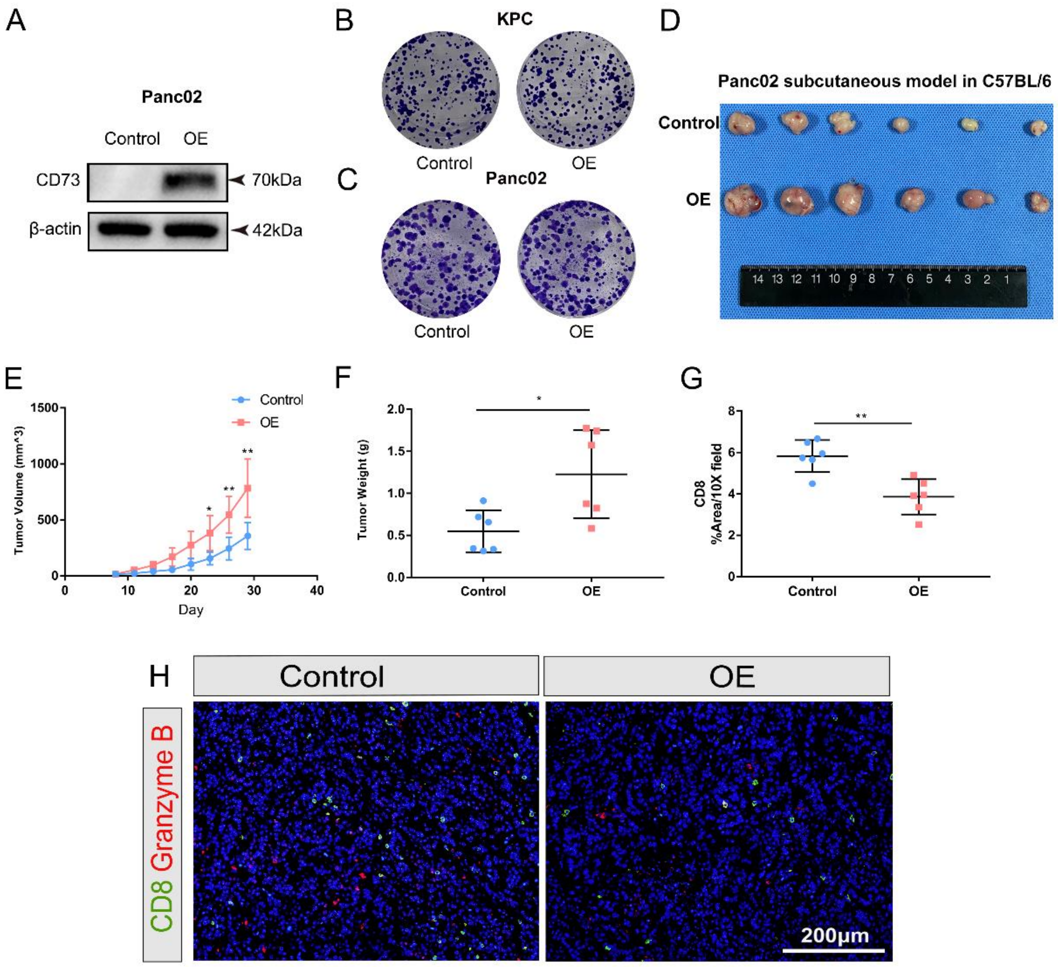

Supplementary Figure 2. Overexpression of CD73 in murine tumor cells promotes tumor growth of subcutaneous tumor-bearing mice. (A) Western blot analysis for CD73 in CD73 overexpressing (OE) and control Panc02 cells. (B-C) Clone formation assay for 2 weeks for cell proliferation after CD73 overexpression in KPC (B) and Panc02 (C) cells. (D-F) Image (D), volumes (E), and weights (F) of the CD73 OE vs Control Panc02 tumors (n=6 samples per group). *$p<0.05$, **$p<0.01$ in Student's t-test. Data presented as mean ± SD. (G) Quantification of immunohistochemistry result of $CD8^+$ T cells in the CD73 OE vs Control Panc02 tumors (n=6 samples per group). **$p<0.01$ in Student's t-test. Data presented as mean ± SD. (H) Representative immunofluorescence staining of CD8 and Granzyme B. Granzyme B: red; CD8: green; DAPI: blue. Scale bar: 200μm.

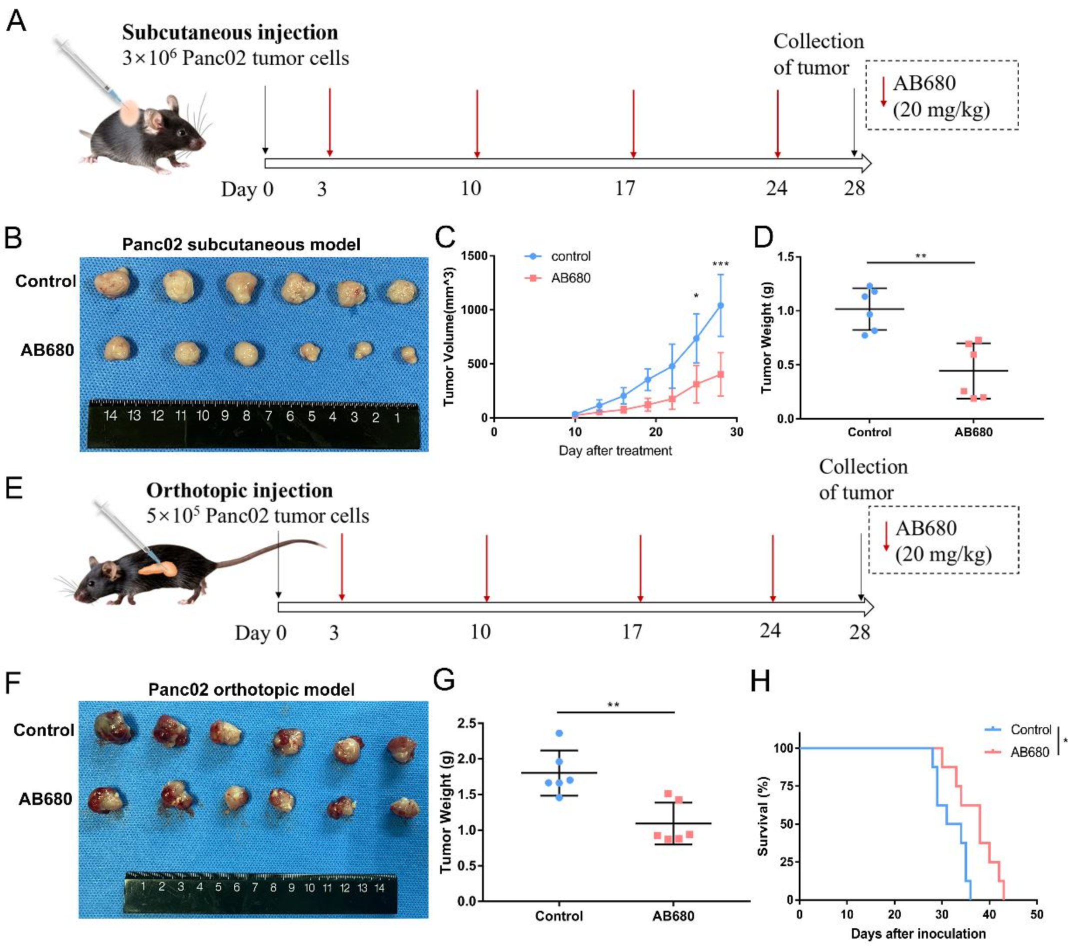

Supplementary Figure 3. AB680 reduces the tumor burden and prolongs survival in Panc02 subcutaneous and orthotopic models. (A) Schematic showing the schedule of CD73 inhibitor AB680 treatment in Panc02 subcutaneous model. (B-D) Image (D), volumes (E), and weights (F) of the AB680-treated vs Control Panc02 subcutaneous tumors (n=6 samples per group). *p<0.05, **p<0.01 in Student's t-test. Data presented as mean ± SD. (E) Schematic showing the schedule of AB680 treatment in Panc02 orthotopic model. (F-G) Image (F) and weights (G) of the AB680-treated vs Control Panc02 orthotopic tumors (n=6 samples per group). **p<0.01 in Student's t-test. Data presented as mean ± SD. (H) Kaplan-Meier survival analysis of tumor-bearing mice untreated or treated with 20 mg/kg AB680 (n=8 samples per group). *p<0.05 in the log-rank test.

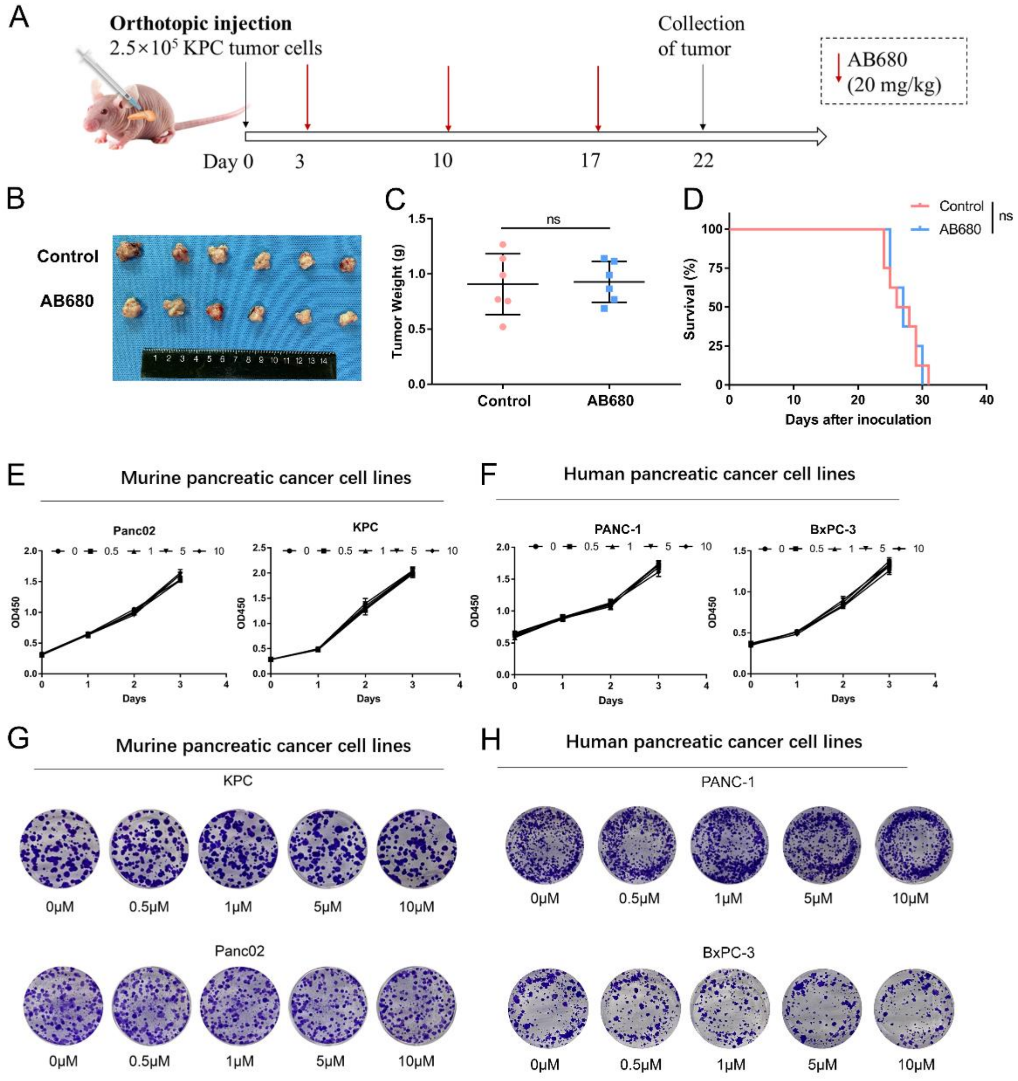

Supplementary Figure 4. AB680 has no anti-tumor effect in nude mice and *in vitro*. (A) Schematic showing the schedule of CD73 inhibitor AB680 treatment in KPC subcutaneous model of nude mice. (B-C) Image (B) and weights (C) of the AB680-treated vs Control KPC orthotopic tumors in nude mice (n=6 samples per group). Data presented as mean ± SD. ns = no significance in Student's t-test. (D) Kaplan-Meier survival analysis of tumor-bearing nude mice untreated or treated with 20 mg/kg AB680 (n=8 samples per group). ns, no significance in the log-rank test. (E-F) CCK-8 assay at 1, 2, and 3 days for cell proliferation after the indicated concentration of AB680 stimulation in murine (E) and human (F) pancreatic cancer cell lines. n=4 samples per group. (G-H) Clone formation assay at 1, 2, and 3 days for cell proliferation after the indicated concentration of AB680 stimulation in murine (G) and human (H) pancreatic cancer cell lines.

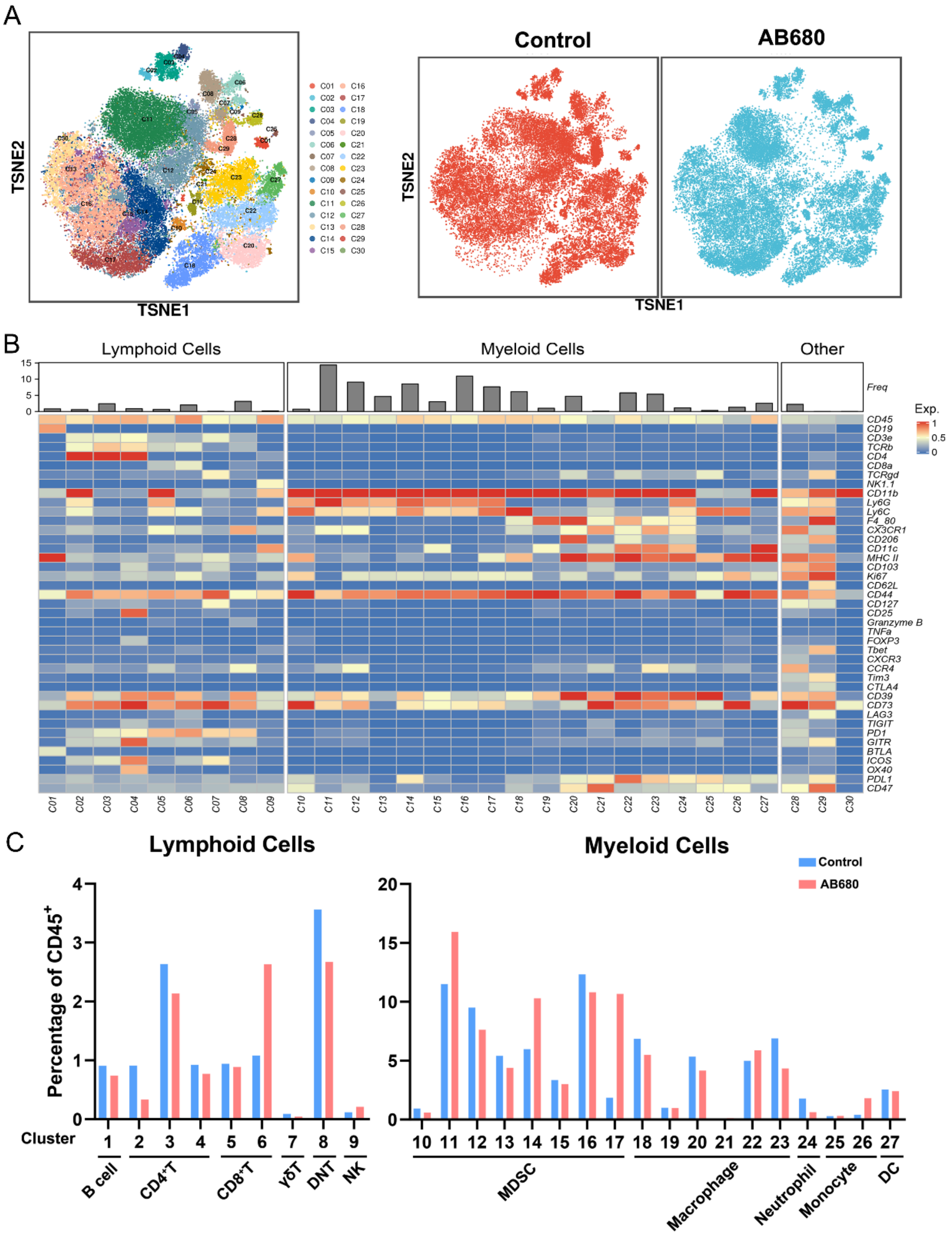

Supplementary Figure 5. AB680 alters the tumor microenvironment. (A) The t-distributed stochastic neighbor embedding (t-SNE) plot of CD45+ tumor-infiltrating immune cells of the total six samples (left), control and AB680-treated samples (right). (B) Heatmap of 42 markers in each cell cluster. (C) The frequencies of each cell cluster among the CD45$^+$ cells in the two groups (n=3 samples per group).

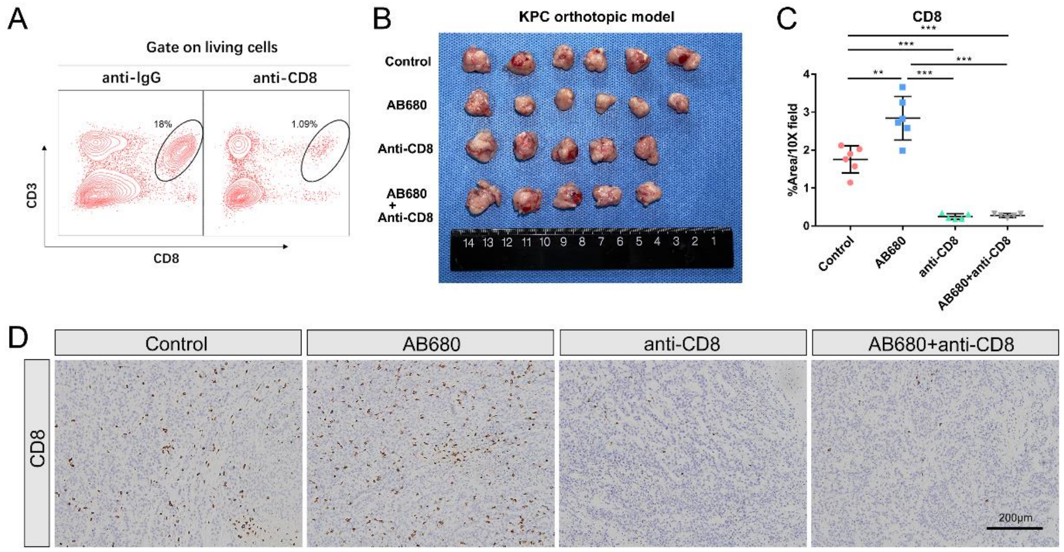

Supplementary Figure 6. $CD8^+$ T cells are the key mediators for the antitumor response of AB680. (A) Flow cytometry analysis showing the depletion of $CD8^+$ T cells in the spleen undergoing treatment with anti-CD8 antibody. (B) The gross appearance of the orthotopic KPC tumors from the Control(n=6), AB680(n=6), anti-CD8(n=5), and AB680+anti-CD8(n=5) groups. (C) Quantification of CD8 % positive area in the tumors of each group at the endpoint. $^{**}p<0.01$, $^{***}p<0.001$ in Student's t-test. Data presented as mean ± SD. (D) Representative images of immunohistochemistry staining of CD8 in the KPC tumors from the control, AB680, anti-CD8, and AB680+anti-CD8 groups. Scale bar: 200μm.

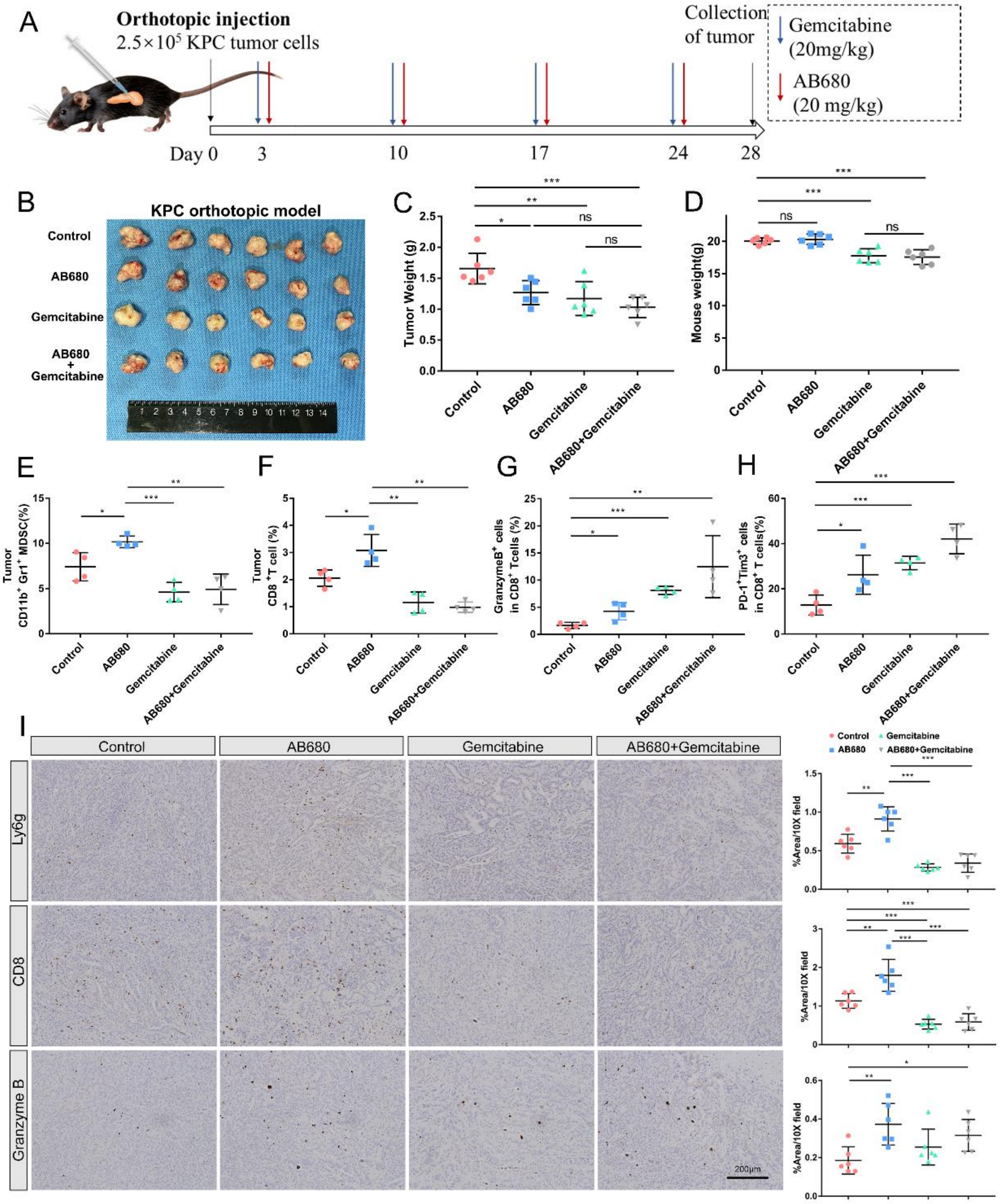

Supplementary Figure 7. Combination of AB680 and gemcitabine cannot enhance the effect to suppress tumor growth. (A) Schematic representation of the therapy schedule for AB680 and gemcitabine in the KPC orthotopic model. (B-D) Image (B), tumor weight (C), and body weight (D) of KPC orthotopic tumors from the indicated treatment groups (n=6 samples per group). Data presented as mean ± SD. ns = no significance, *p<0.05, **p<0.01, ***p<0.001 in Student's t-test. (E-H) The percentages of $CD11b^{+}Gr1^{+}$MDSCs (E), $CD8^{+}$ T cells (F), Granzyme B+ (G), and $PD1^{+}TIM3^{+}$ cells (H) among the total $CD8^{+}$ T cells in the tumors of each group at the endpoint determined by flow cytometry (n=4 samples per group). Data presented as mean ± SD. *p<0.05, **p<0.01, ***p<0.001 in Student's t-test. (I) Representative images and quantification of immunohistochemistry staining of Ly6G, CD8, and Granzyme B in the KPC tumors

from the control, AB680, Gemcitabine, and AB680+Gemcitabine groups. Scale bar: 200μm. n=6 samples per group. Data presented as mean ± SD. *p<0.05, **p<0.01, ***p<0.001 in Student's t-test.

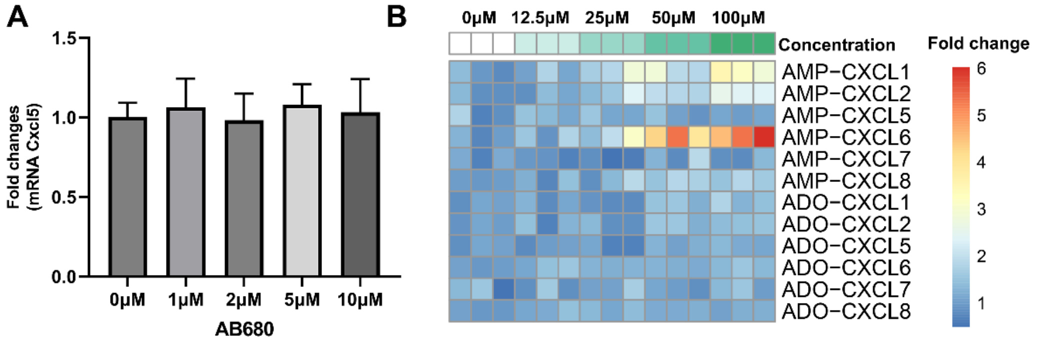

Supplementary Figure 8. Regulation of the indicated chemokine following different concentrations of drugs. (A) qRT-PCR analysis of Cxcl5 mRNA expression in the KPC cells with different concentrations of AB680 at 0, 1, 2, 5, and 10 μM (n=3). (B) qRT-PCR analysis of the ligands of CXCR2 (CXCL1, CXCL2, CXCL5, CXCL6, CXCL7, and CXCL8) mRNA expression in the PANC1 cells with different concentrations of AMP and ADO at 0, 10, 20, 50, and 100 μM (n=3). ADO, adenosine.

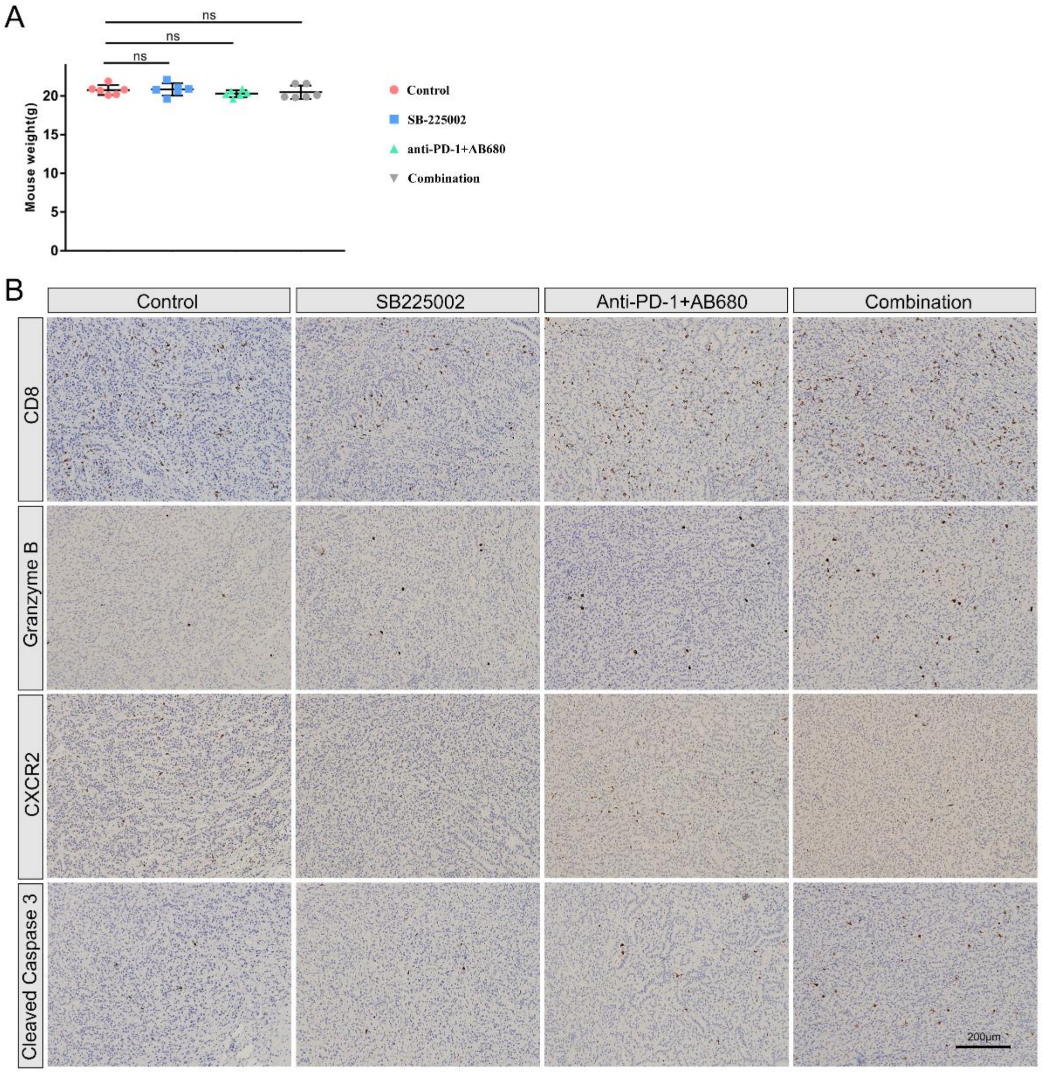

Supplementary Figure 9. CXCR2 inhibitor further potentiates the efficacy of AB680 plus anti-PD-1 therapy. (A) The body weight of the tumor-bearing mice from the indicated groups at the endpoint (n=6 samples per group). Data presented as mean ± SD. ns = no significance in Student's t-test. (B) The representative images of immunohistochemistry staining of CD8, granzyme B, CXCR2, and cleaved caspase 3 in KPC tumors from control, SB252002, anti-PD-1+AB680, and combination groups at the endpoint. Scale bar: 200μm.

Table S1. Information of 42 antibodies used in the CyTOF assay

List	Label	Marker	Clone	Manufacturer	Lot#
**1**	**89Y**	**CD45**	**30-F11**	Biolegend	103102
**2**	**115In**	**CD3 ε**	**145-2C11**	Biolegend	100302
**3**	**139La**	**Ki-67**	**SolA15**	eBioscince	14-5698-82
**4**	**141Pr**	**CD103**	**2E7**	Biolegend	121402
**5**	**142Nd**	**MHC II(I-A/I-E)**	**M5/114.15.2**	Biolegend	107602

**6**	**143Nd**	**CD366(Tim-3)**	**RMT 3-23**	Biolegend	119702
**7**	**144Nd**	**T-bet**	**4B10**	BioLegend	644802
**8**	**145Nd**	**CD152(CTLA-4)**	**UC10-4B9**	Biolegend	106302
**9**	**146Nd**	**CD206(MMR)**	**C068C2**	Biolegend	141702
**10**	**147Sm**	**Ly-6G**	**1A8**	Biolegend	127602
**11**	**148Nd**	**Ly-6C**	**HK1.4**	Biolegend	128002
**12**	**149Sm**	**CD19**	**6D5**	Biolegend	115502
**13**	**150Nd**	**CD127(IL-7Rα)**	**A7R34**	Biolegend	135002
**14**	**151Eu**	**CD62L**	**MEL-14**	Biolegend	104402
**15**	**152Sm**	**CD11c**	**N418**	Biolegend	117302
**16**	**153Eu**	**CD44**	**IM7**	Biolegend	103002
**17**	**154Sm**	**CD73**	**TY/11.8**	Biolegend	127202
**18**	**155Gd**	**CD223 (LAG-3)**	**C9B7W**	Biolegend	125202
**19**	**156Gd**	**GITR(TNFRSF18)**	**2375B**	R&D	MAB52412-100
**20**	**157Gd**	**CD39**	**5F2**	Biolegend	135702
**21**	**158Gd**	**TCR γ/δ**	**GL3**	Biolegend	118140
**22**	**159Tb**	**F4/80**	**C1:A3-1**	BioRAD	MCA497G
**23**	**160Gd**	**CD274(B7-H1,PD-L1)**	**10F.9G2**	Biolegend	124302
**24**	**161Dy**	**TIGIT(VSTM3)**	**2190A**	R&D	MAB72671
**25**	**162Dy**	**CD183(CXCR3)**	**CXCR3-173**	Biolegend	126502
**26**	**163Dy**	**CD25**	**3C7**	Biolegend	101902
**27**	**164Dy**	**CD272**	**6A6**	Biolegend	139117
**28**	**165Ho**	**CD278(ICOS)**	**C398.4A**	BioLegend	313502
**29**	**166Er**	**CD194(CCR4)**	**2G12**	Biolegend	131202
**30**	**167Er**	**CD134(OX40)**	**OX-86**	Biolegend	119429
**31**	**168Er**	**FOXP3**	**FJK-16s**	eBioscince	14-5773-82
**32**	**169Tm**	**CD47**	**Miap301**	Biolegend	127502
**33**	**170Er**	**CD161(NK-1.1)**	**PK136**	Biolegend	108702
**34**	**171Yb**	**CX3CR1**	**SA011F11**	Biolegend	149002
**35**	**172Yb**	**CD279(PD-1)**	**29F.1A12**	Biolegend	135202
**36**	**173Yb**	**Granzyme B Fluidigm**	**GB11**	Fluidigm	3173006B
**37**	**174Yb**	**IFN-γ**	**XMG1.2**	Bio-Xcell	BE0055
**38**	**175Lu**	**TCR β chain**	**H57-597**	Biolegend	109202
**39**	**176Yb**	**TNF-α**	**MP6-XT22**	Biolegend	506302
**40**	**197Au**	**CD4**	**RM4-5**	Biolegend	100520
**41**	**198Pt**	**CD8a**	**53-6.7**	Biolegend	100746
**42**	**209Bi**	**CD11b**	**M1/70**	BioLegend	101202
